# Characterizing population and individual migration patterns among native and restored bighorn sheep (*Ovis canadensis*)

**DOI:** 10.1002/ece3.5435

**Published:** 2019-07-09

**Authors:** Blake Lowrey, Kelly M. Proffitt, Douglas E. McWhirter, Patrick J. White, Alyson B. Courtemanch, Sarah R. Dewey, Hollie M. Miyasaki, Kevin L. Monteith, Julie S. Mao, Jamin L. Grigg, Carson J. Butler, Ethan S. Lula, Robert A. Garrott

**Affiliations:** ^1^ Fish and Wildlife Ecology and Management Program, Department of Ecology Montana State University Bozeman MT USA; ^2^ Montana Department of Fish, Wildlife, and Parks Bozeman MT USA; ^3^ Wyoming Game and Fish Department Jackson WY USA; ^4^ Yellowstone Center for Resources, Yellowstone National Park National Park Service Mammoth WY USA; ^5^ Grand Teton National Park Moose WY USA; ^6^ Idaho Department of Fish and Game Idaho Falls ID USA; ^7^ Haub School of Environment and Natural Resources, Wyoming Cooperative Fish and Wildlife Research Unit, Department of Zoology and Physiology University of Wyoming Laramie WY USA; ^8^ Colorado Parks and Wildlife Glenwood Springs CO USA; ^9^ Colorado Parks and Wildlife Salida CO USA

**Keywords:** augmentation, conservation, individual heterogeneity, migration, migratory diversity, portfolio effects, resource tracking, restoration, translocation

## Abstract

1. Migration evolved as a behavior to enhance fitness through exploiting spatially and temporally variable resources and avoiding predation or other threats. Globally, landscape alterations have resulted in declines to migratory populations across taxa. Given the long time periods over which migrations evolved in native systems, it is unlikely that restored populations embody the same migratory complexity that existed before population reductions or regional extirpation.
2. We used GPS location data collected from 209 female bighorn sheep (*Ovis canadensis*) to characterize population and individual migration patterns along elevation and geographic continuums for 18 populations of bighorn sheep with different management histories (i.e., restored, augmented, and native) across the western United States.
3. Individuals with resident behaviors were present in all management histories. Elevational migrations were the most common population‐level migratory behavior. There were notable differences in the degree of individual variation within a population across the three management histories. Relative to native populations, restored and augmented populations had less variation among individuals with respect to elevation and geographic migration distances. Differences in migratory behavior were most pronounced for geographic distances, where the majority of native populations had a range of variation that was 2–4 times greater than restored or augmented populations.
4. *Synthesis and applications*. Migrations within native populations include a variety of patterns that translocation efforts have not been able to fully recreate within restored and augmented populations. Theoretical and empirical research has highlighted the benefits of migratory diversity in promoting resilience and population stability. Limited migratory diversity may serve as an additional factor limiting demographic performance and range expansion. We suggest preserving native systems with intact migratory portfolios and a more nuanced approach to restoration and augmentation in which source populations are identified based on a suite of criteria that includes matching migratory patterns of source populations with local landscape attributes.

## 1 INTRODUCTION

Seasonal migration has evolved as a complex behavior to enhance fitness and results from interactions between individuals (e.g., learned behavior), their genes, and the environment, notably spatiotemporal variation in resources and interspecific threats (e.g., predation; Dingle & Drake, 2007; Fryxell & Sinclair, 1988; Hebblewhite & Merrill, 2009). Migration is widespread across taxonomic groups and increasingly recognized as fundamental to maintaining populations and communities through effects on population productivity and the lateral transport of nutrients within and across ecosystems (Bolger, Newmark, Morrison, & Doak, 2008; Helfield & Naiman, 2001; Holdo, Holt, Sinclair, Godley, & Thirgood, 2011; Milner‐Gulland, Fryxell, & Sinclair, 2011; Sawyer, Middleton, Hayes, Kauffman, & Monteith, 2016). Moreover, identifying and conserving migration corridors is an important management priority for state (WYGF, 2016) and federal (USDOI, 2018) agencies, and noted as one of the most difficult conservation challenges of the 21st century (Berger, 2004).

Globally, habitat loss, barriers along migratory routes, overexploitation, and climate change have resulted in steep declines of migratory behavior, and for many species, subsequent population declines (Bolger et al., 2008; Milner‐Gulland et al., 2011; Wilcove & Wikelski, 2008). The loss of migration spans nearly all taxonomic groups and has important implications across multiple biological levels of organization as well as direct relevance to economic and social concerns (Harris, Thirgood, Hopcraft, Cromsigt, & Berger, 2009; Wilcove, 2010). Once lost, restoring migrations has been met with limited success, as the source of the initial extirpation (e.g., habitat loss or fragmentation) can persist on the landscape (Wilcove, 2010). Although a few hopeful examples have shown some capacity to restore migrations after mitigating impediments to animal movement, the gains generally come at high economic costs and represent a diminished resemblance of historic migratory patterns (Bartlam‐Brooks, Bonyongo, & Harris, 2011; Ellis et al., 2003).

Bighorn sheep (*Ovis canadensis*) are an iconic mountain ungulate that occur throughout western North America but have struggled to rebound to historic numbers and distributions after overharvest and the introduction of non‐native respiratory pathogens from domestic livestock (Buechner, 1960; Cassirer et al., 2017). While restoration efforts have resulted in modest increases in abundance and distribution, bighorn sheep occupy a small fraction of their former range and occur predominantly in restored populations that number fewer than 100 individuals (Buechner, 1960; Singer, Papouchis, & Symonds, 2000). Throughout their range, previous studies have documented varied migratory behaviors from resident to long‐distant migrants involving all or a subset of individuals within a population (i.e., partial migration; Hurley, 1985; Woolf, O'Shea, & Gilbert, 1970; Martin, 1985; DeCesare & Pletscher, 2006; Sawyer et al., 2016; Courtemanch, Kauffman, Kilpatrick, & Dewey, 2017). Migratory movements clearly influence other large ungulates (Bolger et al., 2008; Sawyer, Kauffman, Nielson, & Horne, 2009; Tucker et al., 2018; White, Davis, Barnowe‐Meyer, Crabtree, & Garrott, 2007) and are positively associated with restoration success (Singer et al., 2000), yet our current understanding of bighorn sheep migration largely stems from management surveys or limited tracking of animals instrumented with VHF collars sampled from single populations.

Bighorn sheep are particularly interesting for studies of migration because of the widespread use of translocations as a management strategy to expand distributions into historic ranges and augment existing populations (Singer et al., 2000; Wild Sheep Working Group, 2015). As of 2015, nearly 1,500 restoration efforts resulted in the translocation of more than 21,500 bighorn sheep in North America (Brewer et al., 2014). Recent comparisons across restored and native populations of bighorn sheep indicate that migration is likely socially learned and culturally transmitted (Jesmer et al., 2018). Restored populations containing individuals that were translocated into novel environments were less migratory than native populations that had maintained a continuous presence on the landscape and developed population “knowledge” of the surrounding environment (Jesmer et al., 2018). These findings contribute important insights regarding the evolution of migration in ungulates, yet population and individual migratory patterns across the varied histories (e.g., restored, augmented, native) are largely undescribed.

We used GPS location data to describe population and individual migration patterns along elevation and geographic gradients among native, augmented, and restored bighorn sheep populations across the western United States. We predicted that the differences in landscape “knowledge” between management histories (e.g., restored, augmented, native) would result in population and individual differences in migration behaviors. Native populations embody a longer period over which generations have had the opportunity to discover and exploit landscape resources, and develop multiple migratory behaviors across varied spatial scales that confer similar individual fitness. Consequently, we hypothesized that the continuous inhabitance of native populations would result in longer migrations over elevation and geographic continuums with more variation in migratory patterns among individuals. In contrast, we hypothesized that migrations within augmented and restored populations would be limited with respect to elevation and geographic distances and exhibit less individual variation in migratory patterns. Our approach represents a broad empirical characterization of seasonal migration in bighorn sheep and provides an evaluation of translocation efforts in restoring seasonal migrations in areas where bighorn sheep were locally extirpated or greatly reduced.

## 2 MATERIALS AND METHODS

### 2.1 Study areas

**Figure 1:**
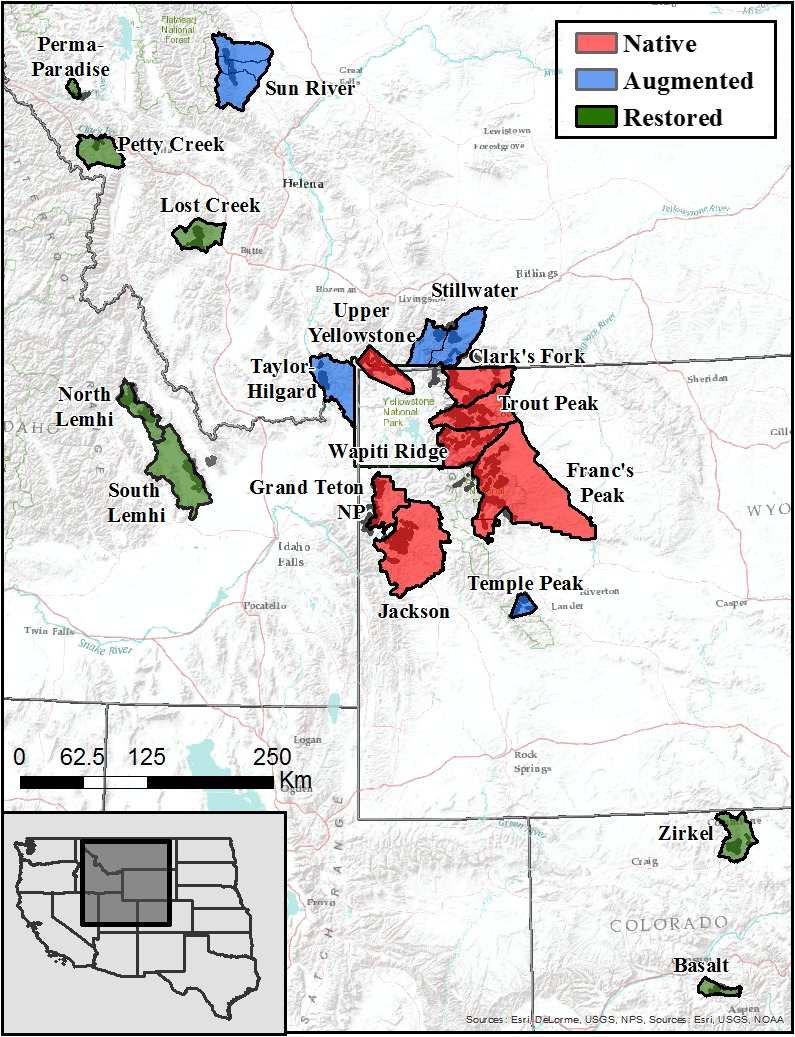
Native (red; *N* = 7), augmented (blue; *N* = 4), and restored (green; *N* = 7) population units used to characterize female bighorn sheep migration patterns, Montana, Wyoming, Idaho, and Colorado, USA, 2008−2017

Our study populations were broadly distributed across Montana, Wyoming, Idaho, and Colorado in the western United States (Figure 1). Within each state, we used winter capture locations to group individuals into population units, which generally adhered to regional management units (i.e., state hunting districts or national park boundaries; Appendix S1). We used population histories to classify study populations as native, augmented, or restored (Table 1). Native populations were never extirpated or augmented and maintained a constant evolutionary history on the landscape. Augmented populations retained a native component that was bolstered through translocations because of concerns over long‐term persistence and low abundance. Population estimates for the remnant native component prior to receiving translocations are not well documented, but generally represent a greatly reduced relic of historic distribution and abundance (Montana Fish Wildlife & Parks, 2010). Restored populations were within historic bighorn sheep range, but created through translocations after the native component was extirpated. For restored or augmented study populations, the cause of the initial extirpation or decline was not specifically documented. Nonetheless, the introduction of exotic pathogens from domestic animals, competition with domestic livestock, and overharvest are widely cited as the known mechanisms resulting in the drastic declines in regional bighorn sheep distribution and abundance in the early‐ to mid‐1900s (Buechner, 1960; Montana Fish, Wildlife, & Parks, 2010; Singer et al., 2000). There are no records indicating the loss of migratory routes as an initial cause of decline in any study population.

**Table 1:** Summary information for the study populations, Montana, Wyoming, Idaho, and Colorado, USA, 2008−2017

State	Population units					Translocation history			
	Name	*N*	Management units^a^	Population estimate^b^	Population type	Year	Number	Source^c^	Migratory behavior of source population
MT	Perma‐Paradise	14	HD‐124	352	Restored	1979	14	WHI	Resident
						2011	22	WHI	Resident
MT	Petty Creek	14	HD‐203	160	Restored	1968	16	MT‐422	Migratory
						1985	4	NBR	Resident
MT	Lost Creek	10	HD‐213	100	Restored	1967	25	MT‐422	Migratory
						1985	2	MT‐121	Migratory
MT	Hilgard	15	HD‐302	280	Augmented	1988	19	MT‐121	Migratory
						1989	5	MT‐121	Migratory
						1989	19	MT‐213	Migratory
						1993	26	WHI	Resident
MT	Sun River	12	HD‐422, 424	150	Augmented	1960	8	MT‐422	Migratory
MT	Stillwater	13	HD‐501, 502	75	Augmented	1968	2	MT‐422	Migratory
						1970	2	MT‐422	Migratory
						1984	3	NBR	Resident
MT	Upper Yellowstone	10	HD‐305, northwest YNP	320	Native	—	—	—	—
WY	Clark's Fork	19	HD‐1, northeast YNP	600	Native	—	—	—	—
WY	Trout Peak	11	HD‐2	700	Native	—	—	—	—
WY	Wapiti Ridge	7	HD‐3	850	Native	—	—	—	—
WY	Franc's Peak	17	HD‐5, 22	840	Native	—	—	—	—
WY	Grand Teton NP^d^	14	GTNP	100	Native	—	—	—	—
WY	Jackson	16	HD‐7	450	Native	—	—	—	—
WY	Temple Peak^d^	8	—	50–75	Augmented	1960	1	WY‐Whiskey	Partial
						1964	20	WY‐Whiskey	Partial
						1965	20	WY‐Whiskey	Partial
						1966	18	WY‐Whiskey	Partial
						1971	13	WY‐Whiskey	Partial
						1972	39	WY‐Whiskey	Partial
						1987	54	WY‐Whiskey	Partial
ID	North Lemhi	9	37A, 29	129	Restored	1986	18	OR‐Lostine	Migratory
						1988	13	ID‐36A	Migratory
						1989	23	ID‐36B	Partial
ID	South Lemhi	6	51, 58	40	Restored	1983	19	WY‐Whiskey	Partial
						1984	22	WY‐Whiskey	Partial
CO	Zirkel	7	S73	120‐130	Restored	2004	26	CO‐S65	Unk
						2005	14	CO‐S65	Unk
CO	Basalt	7	S44	70	Restored	1972	18	CO‐S10	Unk

a The aggregation of management units within each population unit is further described in Appendix S1.

b Estimates were provided by agency management biologists and determined from local knowledge, minimum counts, and recent trends.

c WHI: Wild Horse Island; NBR: National Bison Range; MT, WY, OR, ID, CO: state abbreviations; numbers reference state hunting districts.

d Temple Peak is a nonhunted population without a management unit.

Phenological patterns and landscape heterogeneity are important drivers of migratory behavior in ungulates (Hsiung, Boyle, Cooper, & Chandler, 2018; Merkle et al., 2016; Smolko, Kropil, Pataky, Veselovská, & Merrill, 2018) and were similar across all study areas (Appendix S2). All populations were located in contiguous mountainous landscapes within temperate latitudes and experienced strong seasonal variation in annual climate and spatiotemporal variation in resource availability and quality. Land ownership was dominated by federally managed lands with nearly all populations within or directly adjacent to designated Wilderness areas or National Parks. Winter months were characterized by cold temperatures with moisture predominantly occurring as snow, whereas summer was characterized by relatively warm temperatures with plant phenology advancing from low to high elevations. All study areas experienced green waves of newly emergent vegetation that advanced from low to high elevations over a 2‐month period and a minimum of 1,360 m of topographic relief (Appendix S2). High elevations contained alpine and subalpine flora, mid‐elevations were predominantly characterized by mixed‐coniferous forests, and low elevations consisted of a mosaic of shrub communities and agriculture production.

Estimates of population size varied across the three management histories with native populations being larger than restored or augmented populations on average (Appendix S3). Translocation histories also varied among restored and augmented populations. On average, augmented populations received more translocated individuals and had more translocation events than restored populations, although there was notable variability in the translocation histories among augmented populations (Appendix S3). In addition, the number of years since animals were initially translocated is an important population characteristic in the context of learned migration. Restored and augmented populations had similar translocation timing with an average of 34 (*SD* = 12.7) and 46 (*SD* = 12.3) years, respectively, since the initial translocation (Appendix S3). The use of migratory or partially migratory source populations was the most common translocation strategy (Appendix S3).

All populations contained a suite of native carnivore species, including black bears (*Ursus americanus*), coyotes (*Canis latrans*), mountain lions (*Puma concolor*), bobcats (*Lynx rufus*), and golden eagles (*Aquila chrysaetos*). Excluding Colorado, Idaho, and the Petty Creek and Lost Creek populations in Montana, grizzly bears (*Ursus arctos horribilis*) were also present. Wolves (*Canis lupus*) were present in all study areas outside of Colorado. Most bighorn sheep populations were sympatric with one or more additional ungulates, including mule deer (*Odocoileus hemionus*), white‐tailed deer (*Odocoileus virginianus*), elk (*Cervus canadensis*), and mountain goats (*Oreamnos americanus*).

### 2.2 Data collection and seasonal migration characterizations

Animal capture occurred between 2008 and 2017. We used ground darting, drop nets, and helicopter net‐gunning to capture adult (>1 year old) female bighorn sheep, primarily during winter months. Animals were instrumented with store‐on‐board or remote download GPS collars programmed to record locations at varied intervals ranging from 1 to 13 hr. Where metrics were provided by the GPS collar manufacturer, we censored GPS locations with an HDOP > 10 (D'eon & Delparte, 2005) and a horizontal error >100 m. We then randomly selected a single location per animal for each day to ensure an equal fix rate across individuals and populations.

We characterized seasonal migrations between summer and winter core ranges. We defined core ranges using the location data collected from 15 January to 28 February and 15 July to 15 August for winter and summer, respectively. We defined the core periods to ensure that individuals would be within the respective seasonal range and accommodate the varied capture schedules across populations. We censored individuals with fewer than 10 days of GPS locations within either core seasonal period. In the few instances where we had multiple years of data for an individual, we selected core seasonal ranges from the first year's data that included both the winter and summer periods and excluded data from subsequent years. We characterized geographic distance by measuring the Euclidian distance between centroids (mean coordinates) of the GPS locations collected within the respective core seasonal range date interval. We characterized elevational distance as the seasonal difference between the mean elevations of GPS locations within the respective seasonal periods. Lastly, we described population‐level migration using the median elevation and geographic distance and individual variation within a population according to the 10th and 90th percent distribution quantiles among individuals.

## 3 RESULTS

**Figure 2:**
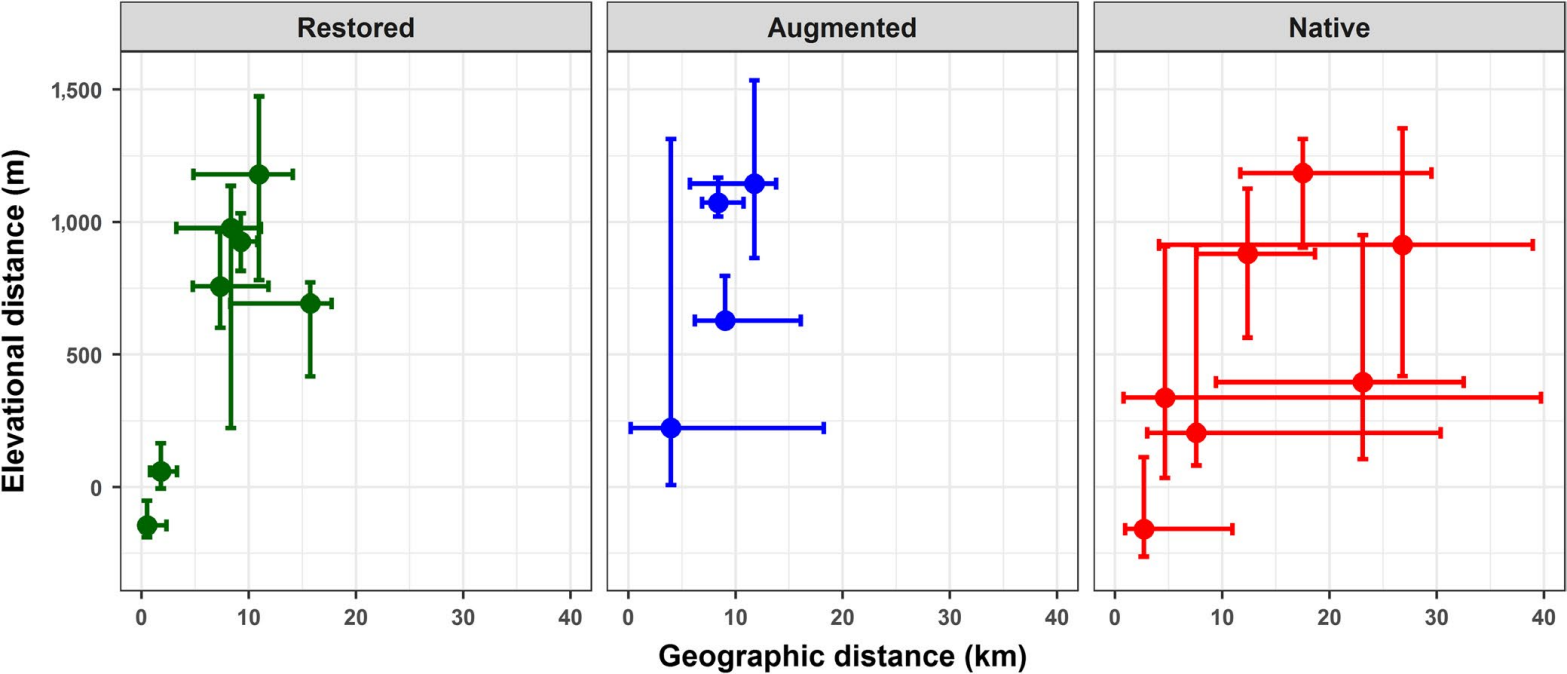
Migration characterizations with respect to elevation and geographic distance between core seasonal ranges for restored (green), augmented (blue), and native (red) populations of female bighorn sheep, in Wyoming, Montana, Idaho, and Colorado, 2008−2017. Closed circles represent population‐level median values. Individual variability is described with the 10th and 90th percent distribution quantiles. Populations with elevation distances below zero had a winter range that was higher than the summer range

We characterized seasonal migrations for 209 female bighorn sheep across 18 populations in four states (Table 1). We obtained data for an average of 12 (range: 6–19) individuals per population with native, augmented, and restored populations well distributed across the range of sample sizes (Table 1 and Appendix S3). Although we generally instrumented slightly more individuals per population in native populations than in restored or augmented populations (Table 1 and Appendix S3), the slight differences in sample sizes across the management histories did not influence our results (Appendix S4). Resident individuals with little to no elevation and geographic distance between core seasonal ranges occurred in all three management histories. Seasonal migrations that spanned elevation gradients (i.e., elevational migrations) were the most common migratory behavior with an average elevation difference of 521 m (±504 *SD*), 840 m (±345 *SD*), and 484 m (±413 *SD*) for restored, augmented, and native populations, respectively. Native populations had a greater range of population‐level elevational migrations, which occurred over longer geographic distances in many populations (Figure 2). The average geographic migration distances were 6.5 km (±5.1 *SD*), 8.7 km (±2.5 *SD*), and 12.4 km (±8.2 *SD*) for restored, augmented, and native populations, respectively. While 15 and 11 km marked the near‐maximum geographic distance of migration for restored and augmented populations, native populations tended to move over longer geographic distances, including a maximum median distance of 27 km (Figure 2).

**Figure 3:**
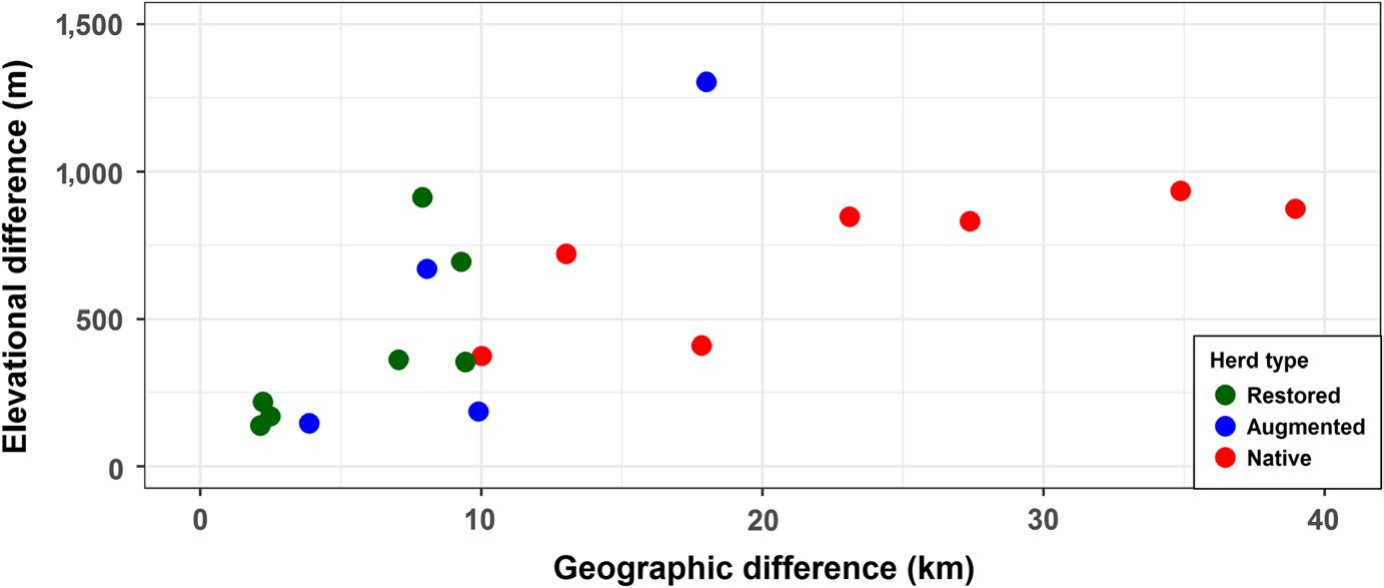
Range of variation in elevation and geographic distances among individuals within each of the 18 restored, augmented, and native bighorn sheep populations, Wyoming, Montana, Idaho, and Colorado, 2008−2017. Each point represents the difference between the 90th and 10th percent quantile for restored (green), augmented (blue), and native (red) populations of female bighorn sheep

There were notable differences in individual variation within a population among the three management histories. As predicted, relative to native populations, restored and augmented populations had less variation among individuals with respect to elevation and geographic distance (Figures 2 and 3). The differences were most pronounced for geographic distances, where the majority of native populations had a range of variation between the 90th and 10th percent distribution quantiles that was 2–4 times greater than in restored or augmented populations (Figure 3 and Table 2). Moreover, individual migrations in native populations spanned a continuum of elevation and geographic distances. In contrast, rather than reflect a continuum of migratory behavior, the limited variation in restored and augmented populations was driven largely by the resident and migrant behaviors characteristic of partially migratory populations (Figure 2 and Appendix S5).

**Table 2:** Average (± *SD*) range of variation for restored, augmented, and native management histories, Montana, Wyoming, Idaho, and Colorado, USA, 2008−2017

Management history	Average (± *SD*) range of variation	
	Elevation (m)	Geography (km)
Restored	355.08 (262.05)	5.00 (3.18)
Augmented	491.13 (428.02)	8.86 (4.76)
Native	691.61 (210.65)	23.12 (10.85)

Note

The range of variation represents the difference between the 90th and 10th percent distribution quantiles for elevation and geographic migration distances averaged over all populations within a management history.

## 4 DISCUSSION

Our study presents a novel and broadscale characterization of population and individual migration behaviors of bighorn sheep from restored, augmented, and native populations using metrics of elevation and geographic distance between seasonal ranges. Although elevational migrations were common among all management histories, there was variation in the distances over which elevational migrations occurred. Migrations in native populations occurred over relatively long geographic distances and were characterized by appreciable variation among individuals along both distance continuums and a range of variation that was up to four times greater than restored or augmented populations. In contrast, the migrations within restored and augmented populations were shorter, especially with respect to geographic distance, and had notably less variation among individuals within a population. While restoration efforts, largely through translocations, have restored elevational migrations in some areas, our results indicate restoration efforts have not successfully restored long‐distance migrations or the migratory diversity observed in native populations.

Within the context of socially learned and culturally transmitted migratory behaviors in ungulates (Jesmer et al., 2018), the landscape “knowledge” of native populations represents the culmination of a long evolutionary history on the landscape. When population knowledge is eliminated or greatly reduced, as in restored or augmented populations, the result is not only a reduction in migratory propensity (Jesmer et al., 2018), but a loss of migratory diversity, inclusive of long‐distance migrations. The successful restoration of elevational migrations may be aided by the “green wave” of newly emergent vegetation which provides an enticing guide from low‐elevation winter ranges to high‐elevation summer ranges (Aikens et al., 2017) and is commonly tracked by large herbivores (Merkle et al., 2016). In contrast, long‐distance migrations that span broad spatial scales and traverse complex landscapes are not easily restored once the historic population knowledge has been lost.

Although the importance of migratory diversity has received little attention in ungulates (but see Morrison, Link, Newmark, Foley, & Bolger, 2011), numerous theoretical and empirical works have highlighted the benefits of migratory diversity across other taxa (Schindler, Armstrong, & Reed, 2015; Webster, Marra, Haig, Bensch, & Holmes, 2002). For example, within anadromous fishes, a portfolio of varied life‐history traits can promote increased resilience, stability, and productivity resulting from the asynchronous dynamics among migratory individuals and reduce risk in a variable environment (Griffiths et al., 2014; Schindler et al., 2010). Similarly, the diffuse spatial arrangement of seasonal ranges in populations with diverse migratory behaviors can increase genetic diversity and population stability in long‐distance avian migrants (Finch, Butler, Franco, & Cresswell, 2016; Webster et al., 2002). While restored and augmented populations of bighorn sheep were able to develop elevational migrations and have some tendency to maintain a partial migration (e.g., a portion of the population migrates), the reduced migratory diversity in these populations may be an additional factor limiting demographic performance. Moreover, because seasonal migration can functionally expand range capacity through behavior (Sawyer et al., 2016), the loss of historic migration patterns in conjunction with poor demographic performance may create a feedback loop where populations remain small with limited range expansion over time.

Given the widespread use of translocations in bighorn sheep management, comparisons among populations with different management histories provided a rare opportunity to evaluate the effectiveness of translocation efforts in restoring migratory patterns and diversity in restored and augmented populations over broad spatial scales. However, although our study areas were similar with respect to many factors that influence migration (Appendices S2 and S3), we were not able to account for all potential differences over our broad study region. For example, local responses to anthropogenic disturbance (Courtemanch et al., 2017; Sawyer et al., 2016), population density (Mysterud et al., 2011), or the migratory behaviors of translocated individuals could all influence migratory diversity. Nonetheless, although the population‐specific mechanisms driving individual variation in migratory behavior are not well understood, increasing migratory diversity may serve as an important objective for ungulate management. Akin to the benefits observed in other taxa, increasing migratory diversity in ungulates may minimize the effects of disease through reducing transmission rates and densities on any single seasonal range (Lowrey et al., 2018; Maichak et al., 2009; Singer, Zeigenfuss, & Spicer, 2001). Moreover, a diffuse distribution also can buffer individuals from other density mediated limits to growth such as interspecific competition and predation (Leech, Jelinski, DeGroot, & Kuzyk, 2017; Lowrey et al., 2018; Singer et al., 2000) as well as stochastic threats such as avalanches (Courtemanch et al., 2017). Maintaining or promoting migratory diversity can also preserve a network of seasonal ranges making populations less reliant on the environmental conditions on any single range (Morrison et al., 2016). At present, while the benefits of migratory diversity have largely been applied to migratory fishes and birds, they provide an intuitive lens with which to view the potential benefits of maintaining and promoting diverse migratory portfolios in terrestrial ungulates.

Migratory behaviors of the source population provide additional insights that can inform translocation strategies and the contemporary assemblage of migratory portfolios. Although the migratory behaviors of translocated individuals are not generally known, migratory behaviors of source populations are often documented through historic reports, VHF monitoring, or GPS collar data. Migratory source populations have been associated with increased restoration success in ungulates (Singer et al., 2000) and were the most common sources among our study populations. We had a limited number of resident source populations and were unable to draw definitive conclusions regarding the effect of migratory behavior of the source population on contemporary migratory diversity. However, with the exception of Petty Creek, all populations that were restored with individuals from migratory sources had a migratory component (Figure S3.6 and Appendix S5). In contrast, Perma‐Paradise was the only population that was restored from an exclusively resident source population, and the translocation effort resulted in a contemporary resident population (Figure S3.6 and Appendix S5). The tendency for ungulates translocated from resident populations to retain their resident behavior rather than develop seasonal migrations when placed in novel mountain environments has been observed in other populations of bighorn sheep, moose (*Alces alces*), and woodland caribou (*Rangifer tarandus caribou*; Jesmer et al., 2018; Leech et al., 2017; Warren, Peek, Servheen, & Zager, 1996) and may lead to reduced demographic performance (Wiedmann & Sargeant, 2014). In addition to forgoing the possible nutritional benefits associated with migration, resident populations are more likely to experience detrimental epizootics resulting from higher pathogen transmission rates on a single year‐round range (Singer et al., 2001). Given the observed benefits of migratory behavior in bolstering restoration success (Singer et al., 2000), we suggest using migratory source populations in ungulate restoration, notwithstanding local management priorities which may situationally favor a resident behavior.

As GPS technology continues to enhance our ability to track and map animal migrations, there are an increasingly large number of seasonal migrations that do not fit within traditional definitions (Dingle & Drake, 2007). Rather than adopt a dichotomous classification (e.g., resident or migrant), seasonal migrations are being increasingly interpreted along a behavioral continuum (Barker, Mitchell, Proffitt, & Devoe, 2018; Cagnacci et al., 2011; Sawyer et al., 2016). Our results expand on this approach through recognizing not only variation in geographic distances, but also variation in elevational distances within and among populations. Evaluating migratory strategies along a continuum may provide additional insights when describing migratory metrics (e.g., timing) or differences in demographic performance among individuals in a population. For example, in addition to examining the ecological (e.g., spatial, temporal, demographic) differences between resident and migratory components of partially migratory populations (Hebblewhite & Merrill, 2009; Middleton et al., 2013; Rolandsen et al., 2016), the characterization of multiple migratory behaviors within a population may help to explain demographic differences among subpopulation components with different migratory behaviors (Barker et al., 2018; Lowrey, 2018; Sawyer et al., 2016).

While nearly a century of bighorn sheep restoration has resulted in modest increases in distribution and abundance, seasonal migrations in restored and augmented populations do not mirror the diversity observed in native populations. Indeed, once lost, diverse migratory portfolios have proven difficult to restore. With the continued increase in ecological threats, our work highlights the importance of preserving native systems with intact migratory portfolios. In addition, we suggest a more nuanced approach to restoration and augmentation in which source populations are identified based on a suite of criteria that includes migration patterns. While disease histories and the presence of respiratory pathogens are becomingly increasingly important in informing translocations and restoration efforts (Butler et al., 2017, 2018), migration patterns of source populations are not often considered, yet are known to support translocation success (Singer et al., 2000). Targeted management experiments that more directly link migration patterns of source populations with landscape attributes in restored areas may be an effective tool to build diversity into restored or augmented ungulate populations (Warren et al., 1996). While individual migratory behaviors are often not known prior to translocations, moving individuals from migratory populations into landscapes with attributes that support migratory behavior (e.g., topographic and phenological heterogeneity) is likely the best option for managers trying to restore populations and bolster migratory diversity. While we recognize residency as a situationally important management priority (e.g., purposely minimizing range expansion), where migratory behavior is desired, we suggest that in addition to increasing abundance and distribution, there is value in simultaneously increasing migratory diversity, and in so doing, building resilience to future perturbations and mirroring the migratory portfolios observed in native populations. Lastly, we encourage work to further elucidate the mechanisms influencing migratory diversity across multiple spatial scales and the potential demographic benefit to ungulates.

## CONFLICT OF INTEREST

None declared.

## AUTHORS' CONTRIBUTIONS

B.L. and R.A.G. conceived the idea and methodological approach; B.L. performed the analysis and wrote the initial draft of the manuscript; and all authors were involved in field efforts to collect and provide data, contributed critically to the manuscript, and gave final approval for publication.

## DATA ACCESSIBILITY

Data supporting the findings of this study are available via DataDryad (https://doi.org/10.5061/dryad.q08jj84).

## References

[ece35435-bib-0001] Aikens, E. O. , Kauffman, M. J. , Merkle, J. A. , Dwinnell, S. P. H. , Fralick, G. L. , & Monteith, K. L. (2017). The greenscape shapes surfing of resource waves in a large migratory herbivore. Ecology Letters, 20, 741–750. 10.1111/ele.12772 28444870

[ece35435-bib-0002] Barker, K. J. , Mitchell, M. S. , Proffitt, K. M. , & Devoe, J. D. (2018). Land management alters traditional nutritional benefits of migration for elk. Journal of Wildlife Management, 83, 167–174.

[ece35435-bib-0003] Bartlam‐Brooks, H. L. A. , Bonyongo, M. C. , & Harris, S. (2011). Will reconnecting ecosystems allow long‐distance mammal migrations to resume? A case study of a zebra *Equus burchelli* migration in Botswana. Oryx, 45, 210–216.

[ece35435-bib-0004] Berger, J. (2004). The last mile: How to sustain long‐distance migration in mammals. Conservation Biology, 18, 320–331. 10.1111/j.1523-1739.2004.00548.x

[ece35435-bib-0005] Bolger, D. T. , Newmark, W. D. , Morrison, T. A. , & Doak, D. F. (2008). The need for integrative approaches to understand and conserve migratory ungulates. Ecology Letters, 11, 63–77. 17897327 10.1111/j.1461-0248.2007.01109.x

[ece35435-bib-0006] Brewer, C. E. , Bleich, V. C. , Foster, J. A. , Hosch‐Hebdon, T. , McWhirter, D. E. , Rominger, E. M. , … Wiedmann, B. P. (2014). Bighorn sheep: Conservation challenges and management strategies for the 21st century. Cheyenne, WY: Wild Sheep Working Group, Western Association of Fish and Wildlife Agencies.

[ece35435-bib-0007] Buechner, H. K. (1960). The bighorn sheep in the United States, its past, present, and future. Wildlife Monographs, 1(4), 3–174.

[ece35435-bib-0008] Butler, C. J. , Edwards, W. H. , Jennings‐Gaines, J. E. , Killion, H. J. , Wood, M. E. , McWhirter, D. E. , … Garrott, R. A. (2017). Assessing respiratory pathogen communities in bighorn sheep populations: Sampling realities, challenges, and improvements. PLoS Biology, 12, e0180689 10.1371/journal.pone.0180689 PMC5510838 28708832

[ece35435-bib-0009] Butler, C. J. , Edwards, W. H. , Paterson, J. T. , Proffitt, K. M. , Jennings‐Gaines, J. E. , Killion, H. J. , … Garrott, R. A. (2018). Respiratory pathogens and their association with population performance in Montana and Wyoming bighorn sheep populations. PLoS Biology, 13, e0207780 10.1371/journal.pone.0207780 PMC6257920 30475861

[ece35435-bib-0010] Cagnacci, F. , Focardi, S. , Heurich, M. , Stache, A. , Hewison, A. J. M. , Morellet, N. , … Urbano, F. (2011). Partial migration in roe deer: Migratory and resident tactics are end points of a behavioral gradient determined by ecological factors. Oikos, 120, 1790–1802.

[ece35435-bib-0011] Cassirer, E. F. , Manlove, K. R. , Almberg, E. S. , Kamath, P. L. , Cox, M. , Wolff, P. , … Besser, T. E. (2017). Pneumonia in bighorn sheep: Risk and resilience. Journal of Wildlife Management, 82, 32–45. 10.1002/jwmg.21309

[ece35435-bib-0012] Courtemanch, A. B. , Kauffman, M. J. , Kilpatrick, S. , & Dewey, S. R. (2017). Alternative foraging strategies enable a mountain ungulate to persist after migration loss. Ecosphere, 8, 1–16. 10.1002/ecs2.1855 29552374

[ece35435-bib-0013] DeCesare, N. J. , & Pletscher, D. H. (2006). Movements, connectivity, and resource selection of Rocky Mountain bighorn sheep. Journal of Mammalogy, 87, 531–538. 10.1644/05-MAMM-A-259R1.1

[ece35435-bib-0014] D'eon, R. G. , & Delparte, D. (2005). Effects of radio‐collar position and orientation on GPS radio‐collar performance, and the implications of PDOP in data screening. Journal of Applied Ecology, 42, 383–388. 10.1111/j.1365-2664.2005.01010.x

[ece35435-bib-0015] Dingle, H. , & Drake, V. A. (2007). What is migration? BioScience, 57, 113–121. 10.1641/B570206

[ece35435-bib-0016] Ellis, D. H. , Sladen, W. J. L. , Lishman, W. A. , Clegg, K. R. , Duff, J. W. , Gee, G. F. , & Lewis, J. C. (2003). Motorized migrations: The future or mere fantasy? BioScience, 53, 260–264. 10.1641/0006-3568(2003)053[0260:MMTFOM]2.0.CO;2

[ece35435-bib-0017] Finch, T. , Butler, S. J. , Franco, A. M. A. , & Cresswell, W. (2016). Low migratory connectivity is common in long‐distance migrant birds. Journal of Animal Ecology, 86, 662–673. 10.1111/1365-2656.12635 28093769

[ece35435-bib-0018] Fryxell, J. M. , & Sinclair, A. R. E. (1988). Causes and consequences of migration by large herbivores. Trends in Ecology & Evolution, 3, 237–241. 10.1016/0169-5347(88)90166-8 21227239

[ece35435-bib-0019] Gilroy, J. J. , Gill, J. A. , Butchart, S. H. M. , Jones, V. R. , & Franco, A. M. A. (2016). Migratory diversity predicts population declines in birds. Ecology Letters, 19, 308–317. 10.1111/ele.12569 26807694

[ece35435-bib-0020] Griffiths, J. R. , Schindler, D. E. , Armstrong, J. B. , Scheuerell, M. D. , Whited, D. C. , Clark, R. A. , … Volk, E. C. (2014). Performance of salmon fishery portfolios across western North America. Journal of Applied Ecology, 51, 1554–1563. 10.1111/1365-2664.12341 25552746 PMC4277685

[ece35435-bib-0021] Harris, G. , Thirgood, S. , Hopcraft, J. G. C. , Cromsigt, J. P. G. M. , & Berger, J. (2009). Global decline in aggregated migrations of large terrestrial mammals. Endangered Species Research, 7, 55–76. 10.3354/esr00173

[ece35435-bib-0022] Hebblewhite, M. , & Merrill, E. H. (2009). Trade‐offs between predation risk and forage differ between migrant strategies in a migratory ungulate. Ecology, 90, 3445–3454. 10.1890/08-2090.1 20120812

[ece35435-bib-0023] Helfield, J. M. , & Naiman, R. J. (2001). Effects of salmon‐derived nitrogen on riparian forest growth and implications for stream productivity. Ecology, 82, 2403–2409. 10.1890/0012-9658(2001)082[2403:EOSDNO]2.0.CO;2

[ece35435-bib-0024] Holdo, R. M. , Holt, R. D. , Sinclair, A. R. , Godley, B. J. , & Thirgood, S. (2011). Migration impacts on communities and ecosystems: Empirical evidence and theoretical insights In Milner‐Gulland E. J., Fryxell J. M., & Sinclair A. R. E. (Eds.), Animal migration: A synthesis (pp. 131–143). Oxford, NY: Oxford University Press.

[ece35435-bib-0025] Hsiung, A. C. , Boyle, W. A. , Cooper, R. J. , & Chandler, R. B. (2018). Altitudinal migration: Ecological drivers, knowledge gaps, and conservation implications. Biological Reviews, 93, 2049–2070. 10.1111/brv.12435 29877015

[ece35435-bib-0026] Hurley, K. P. (1985). The trout peak bighorn sheep herd, Northwestern Wyoming. Thesis. Laramie, WY: The University of Wyoming.

[ece35435-bib-0027] Jesmer, B. R. , Merkle, J. A. , Goheen, R. J. , Aikens, E. O. , Beck, J. L. , Courtemanch, A. B. , … Kauffman, M. J. (2018). Is ungulate migration culturally transmitted? Evidence of social learning from translocated animals. Science, 361, 1023–1025. 10.1126/science.aat0985 30190405

[ece35435-bib-0028] Leech, H. , Jelinski, D. E. , DeGroot, L. , & Kuzyk, G. (2017). The temporal niche and seasonal differences in predation risk to translocated and resident woodland caribou (*Rangifer tarandus caribou*). Canadian Journal of Zoology, 95, 809–820.

[ece35435-bib-0029] Lowrey, B. (2018). Spatial ecology of mountain ungulates in the northern Rocky Mountains: Range expansion, habitat characteristics, niche overlap, and migratory diversity. Ph.D. Dissertation. Fish and Wildlife Biology, Montana State University, Montana, USA.

[ece35435-bib-0030] Lowrey, B. , Butler, C. J. , Edwards, W. H. , Wood, M. E. , Dewey, S. R. , Fralick, G. L. , … Garrott, R. A. (2018). A survey of bacterial respiratory pathogens in native and introduced mountain goats (*Oreamnos americanus*). Journal of Wildlife Diseases, 54, 852–858. 29902131 10.7589/2018-02-025

[ece35435-bib-0031] Maichak, E. J. , Scurlock, B. M. , Rogerson, J. D. , Meadows, L. L. , Barbknecht, A. E. , Edwards, H. E. , & Cross, P. C. (2009). Effects of management, behavior, and scavenging on risk of brucellosis transmission in elk of western Wyoming. Journal of Wildlife Diseases, 45, 398–410. 19395749 10.7589/0090-3558-45.2.398

[ece35435-bib-0032] Martin, S. A. (1985). Ecology of the Rock Creek bighorn sheep population, Beartooth Mountains. Thesis. Bozeman, Montana: Montana State University.

[ece35435-bib-0033] Merkle, J. A. , Monteith, K. L. , Aikens, E. O. , Hayes, M. M. , Hersey, K. R. , Middleton, A. D. , … Kauffman, M. J. (2016). Large herbivores surf waves of green‐up during spring. Philosophical Transactions of the Royal Society B: Biological Sciences, 283, 1–8. 10.1098/rspb.2016.0456 PMC4936031 27335416

[ece35435-bib-0034] Middleton, A. D. , Kauffman, M. J. , McWhirter, D. E. , Cook, J. G. , Cook, R. C. , Nelson, A. A. , … Klaver, R. W. (2013). Animal migration amid shifting patterns of phenology and predation: Lessons from a Yellowstone elk population. Ecology, 94, 1245–1256. 23923485 10.1890/11-2298.1

[ece35435-bib-0035] Milner‐Gulland, E. J. , Fryxell, J. M. , & Sinclair, A. R. E. (2011). Animal migration: A synthesis. Oxford, NY: Oxford University Press.

[ece35435-bib-0036] Montana Fish, Wildlife & Parks (2010). Montana bighorn sheep conservation strategy. Montana Fish, Wildlife & Parks. Retrieved from http://fwp.mt.gov/fishAndWildlife/management/bighorn/plan.html

[ece35435-bib-0037] Morrison, T. A. , Link, W. A. , Newmark, W. D. , Foley, C. A. H. , & Bolger, D. T. 2011.

[ece35435-bib-0038] Morrison, T. A. , Link, W. A. , Newmark, W. D. , Foley, C. A. H. , & Bolger, D. T. (2016). Tarangire revisited: Consequences of declining connectivity in a tropical ungulate population. Biological Conservation, 197, 53–60.

[ece35435-bib-0039] Mysterud, A. , Egil, E. L. , Zimmermann, B. , Bischof, R. , Veiberg, V. , & Meisingset, E. (2011). Partial migration in expanding red deer populations at northern latitudes – A role for density dependence? Oikos, 120, 1817–1825. 10.1111/j.1600-0706.2011.19439.x

[ece35435-bib-0040] Rolandsen, C. M. , Solberg, E. J. , Sæther, B.‐E. , Moorter, B. V. , Herfindal, I. , & Bjørneraas, K. (2016). On fitness and partial migration in a large herbivore – migratory moose have higher reproductive performance than residents. Oikos, 126, 547–555. 10.1111/oik.02996

[ece35435-bib-0041] Sawyer, H. , Kauffman, M. J. , Nielson, R. M. , & Horne, J. S. (2009). Identifying and prioritizing ungulate migration routes for landscape‐level conservation. Ecological Applications, 19, 2016–2025. 10.1890/08-2034.1 20014575

[ece35435-bib-0042] Sawyer, H. , Middleton, A. D. , Hayes, M. M. , Kauffman, M. J. , & Monteith, K. L. (2016). The extra mile: Ungulate migration distance alters the use of seasonal range and exposure to anthropogenic risk. Ecosphere, 7, 1–11. 10.1002/ecs2.1534

[ece35435-bib-0043] Schindler, D. E. , Armstrong, J. B. , & Reed, T. E. (2015). The portfolio concept in ecology and evolution. Frontiers in Ecology and the Environment, 13, 257–263. 10.1890/140275

[ece35435-bib-0044] Schindler, D. E. , Hilborn, R. , Chasco, B. , Boatright, C. P. , Quinn, T. P. , Rogers, L. A. , & Webster, M. S. (2010). Population diversity and the portfolio effect in an exploited species. Nature, 465, 609–612. 10.1038/nature09060 20520713

[ece35435-bib-0045] Singer, F. J. , Papouchis, C. M. , & Symonds, K. K. (2000). Translocations as a tool for restoring populations of bighorn sheep. Restoration Ecology, 8, 6–13. 10.1046/j.1526-100x.2000.80061.x

[ece35435-bib-0046] Singer, F. J. , Zeigenfuss, L. C. , & Spicer, L. (2001). Role of patch size, disease, and movement in rapid extinction of bighorn sheep. Conservation Biology, 15, 1347–1354. 10.1046/j.1523-1739.2001.99488.x

[ece35435-bib-0047] Smolko, P. , Kropil, R. , Pataky, T. , Veselovská, A. , & Merrill, E. (2018). Why do migrants move downhill? The effects of increasing predation and density on red deer altitudinal migration in temperate Carpathian forests. Mammal Research, 63, 297–305. 10.1007/s13364-018-0355-3

[ece35435-bib-0048] Tucker, M. A. , Böhning‐Gaese, K. , Fagan, W. F. , Fryxell, J. M. , Van Moorter, B. , Alberts, S. C. , … Mueller, T. (2018). Moving in the Anthropocene: Global reductions in terrestrial mammalian movements. Science, 359, 466–469. 10.1126/science.aam9712 29371471

[ece35435-bib-0049] United States Department of Interior (USDOI) (2018). Secretarial order 3362: Improving habitat quality in Western big game winter range and migration corridors. Retrieved from https://www.doi.gov/sites/doi.gov/files/uploads/so_3362_migration.pdf

[ece35435-bib-0050] Warren, C. D. , Peek, J. M. , Servheen, G. L. , & Zager, P. (1996). Habitat use and movements of two ecotypes of translocated Caribou in Idaho and British Columbia. Conservation Biology, 10, 547–553. 10.1046/j.1523-1739.1996.10020547.x

[ece35435-bib-0051] Webster, M. S. , Marra, P. P. , Haig, S. M. , Bensch, S. , & Holmes, R. T. (2002). Links between worlds: Unraveling migratory connectivity. Trends in Ecology & Evolution, 17, 76–83. 10.1016/S0169-5347(01)02380-1

[ece35435-bib-0052] White, P. J. , Davis, T. L. , Barnowe‐Meyer, K. K. , Crabtree, R. L. , & Garrott, R. A. (2007). Partial migration and philopatry of Yellowstone pronghorn. Biological Conservation, 135, 502–510. 10.1016/j.biocon.2006.10.041

[ece35435-bib-0053] Wiedmann, B. P. , & Sargent, G. A. (2014). Ecotypic variation in recruitment of reintroduced bighorn sheep: Implications for translocation. The Journal of Wildlife Management, 78, 394–401.

[ece35435-bib-0054] Wilcove, D. S. (2010). No way home: The decline of the world's great animal migrations (3rd ed.). Washington, DC: Island Press.

[ece35435-bib-0055] Wilcove, D. S. , & Wikelski, M. (2008). Going, going, gone: Is animal migration disappearing? PLoS Biology, 6, e188 10.1371/journal.pbio.0060188 18666834 PMC2486312

[ece35435-bib-0056] Wild Sheep Working Group (2015). Records of wild sheep translocations: United States and Canada, 1922‐present. Cheyenne, WY: Western Association of Fish and Wildlife Agencies.

[ece35435-bib-0057] Woolf, A. , O'Shea, T. , & Gilbert, D. L. (1970). Movements and behavior of bighorn sheep on summer ranges in Yellowstone National Park. Journal of Wildlife Management, 34, 446–450. 10.2307/3799031

[ece35435-bib-0058] Wyoming Game and Fish Department (WYGF) (2016). Wyoming Game and Fish Department Ungulate Migration Corridor Strategy. Retrieved from https://wgfd.wyo.gov/WGFD/media/content/PDF/Habitat/Habitat%20Information/Ungulate-Migration-Corridor-Strategy_Final_020416.pdf

