# Supplementary material for: Characterizing population and individual migration patterns among native and restored bighorn sheep (Ovis canadensis)
Source: Ecol Evol. 2019 Jul 9;9(15):8829–39. doi: 10.1002/ece3.5435 (PMC6686647; doi:10.1002/ece3.5435)
Supplement: Supplementary file 1 [file ECE3-9-8829-s001.docx]

**Appendix S1: Detailed description of the population delineations**

The delineation of populations to describe seasonal migration patterns and variability was a critical step in our analysis that highlighted the importance of scale in ecology (Wiens 1989). While we recognize the scale-dependence of our results (e.g. Levin 1992), we feel the populations detailed herein are an appropriate aggregate based on a number of factors. We delineated populations using regional management units (i.e. hunting districts or national park boundaries) which provided a similar grouping system that could be applied across the broad study region. Moreover, management units are the most relevant administrative unit for regional managers and are often used to summarize other population attributes (i.e. vital rates, abundance, disease prevalence, etc.). By linking our work with management units, our results are directly applicable to regional management efforts and represent the same spatial scale.

We grouped individual bighorn sheep into populations based on the capture location. In most instances, the population was synonymous with local management units, although there were a few exceptions where we aggregated adjacent management units and/or individuals to better reflect groupings based on the GPS locations and local geography. We recognize the subjective nature of these decisions and have provided detailed descriptions of the areas where aggregation occurred. There was no lumping within the Colorado or Idaho population units.

**Montana populations**

Perma-Paradise (HD-124), Petty Creek (HD-203), Lost Creek (HD-213), and Taylor-Hilgard (HD-302) populations were all characterized by a single management unit (Fig S1.1). The Sun River population was an aggregate of individuals captured in HD-424 and HD-422, both of which had 6 animals. The adjacent management units (HD-421 and HD-423) were also used by instrumented bighorn sheep (Fig S1.1). There were four individuals captured in the northwest corner of Yellowstone National Park (YNP), roughly 9 km from the animals within the Upper Yellowstone (HD-305). We aggregated animals captured in northwest YNP with those in Upper Yellowstone (HD-305) to make a single population with 10 individuals. All animals in Stillwater were captured within a single management unit (HD-502) but separated into two adjacent units (HD-501) in summer (Fig S1.1). These animals were considered a single population with 13 individuals.

**Wyoming populations**

Animals in Wyoming were broadly distributed across the Absaroka Mountains with additional discontinuous populations in the Teton, Gros Ventre, and Wind River ranges (Fig S1.2). The Clark’s Fork population contained a total of 19 collared individuals, which included two animals captured within the northeast corner of YNP and another single individual capture roughly 2 km north of the MT-WY border as this is a “shared” or trans-boundary herd. While the individual was captured in MT, we included it within Wyoming’s Clark’s Fork herd (Fig S1.2). The Trout Peak (HD-2) and Wapiti Ridge (HD-3) populations are separated by the North Fork of the Shoshone River. These herds share some common winter ranges, and in one instance an individual captured in HD-2 was more strongly associated with HD-3 based on GPS locations. Although the capture location was within HD-2, we grouped the individual with HD-3, the Wapiti Ridge population (Fig S1.2). The Franc’s Peak population included 17 collared individuals, of which three were captured in HD-22, the management unit adjacent to HD-5 to the southwest. Lastly, Temple Peak is not a hunted herd and does not have a designated management unit but was aggregated as a single herd with 8 collard individuals. The remaining populations, Jackson (HD-7) and Grand Teton NP were characterized by a single management unit.


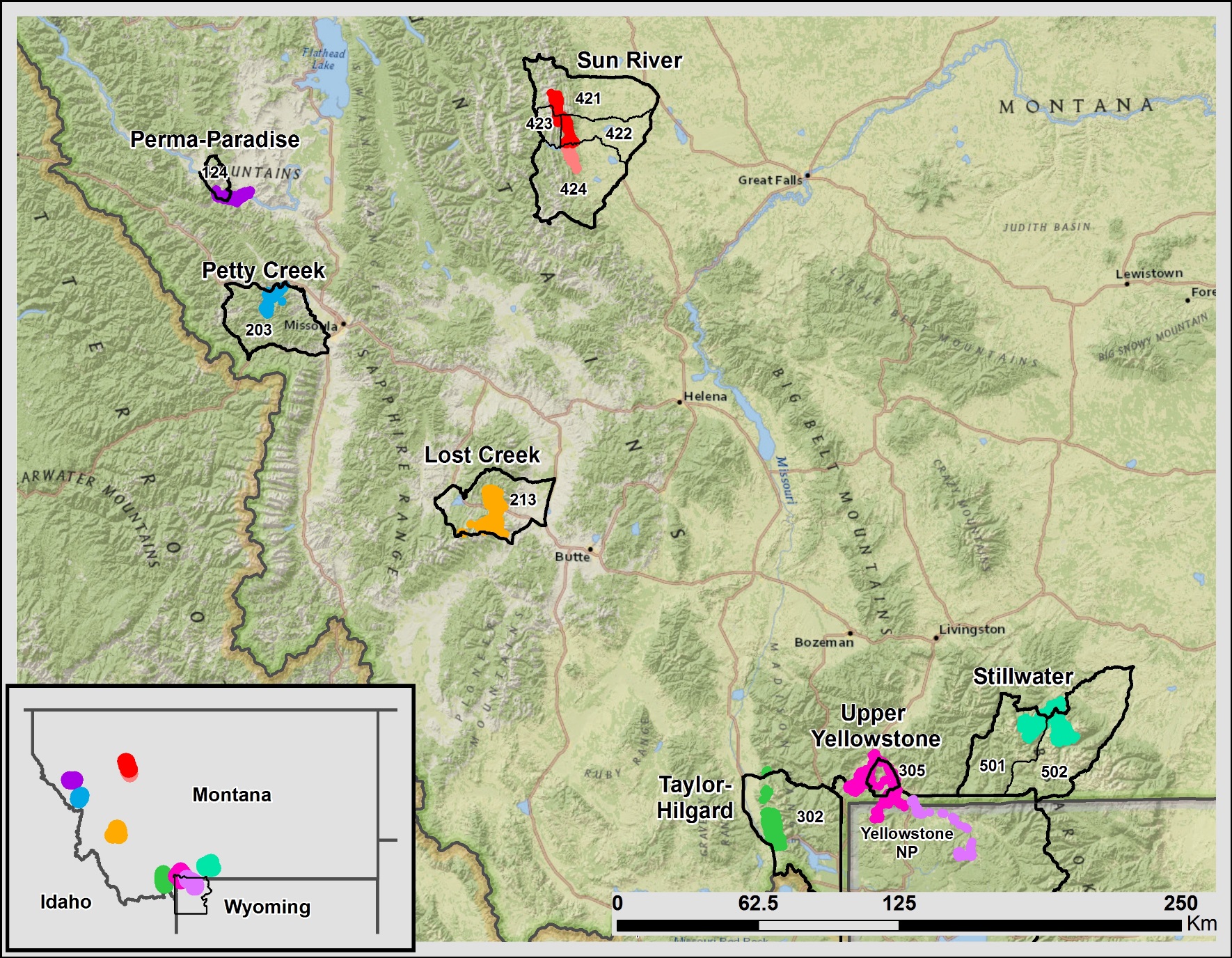


**Fig S1.1** Montana herd units. Within the Sun River herd unit we aggregated individuals captured within HD-424 (pink) and HD-422 (red). Within the Upper Yellowstone herd unit we aggregated individuals captured in HD-305 (dark pink) and northwest YNP (light pink).


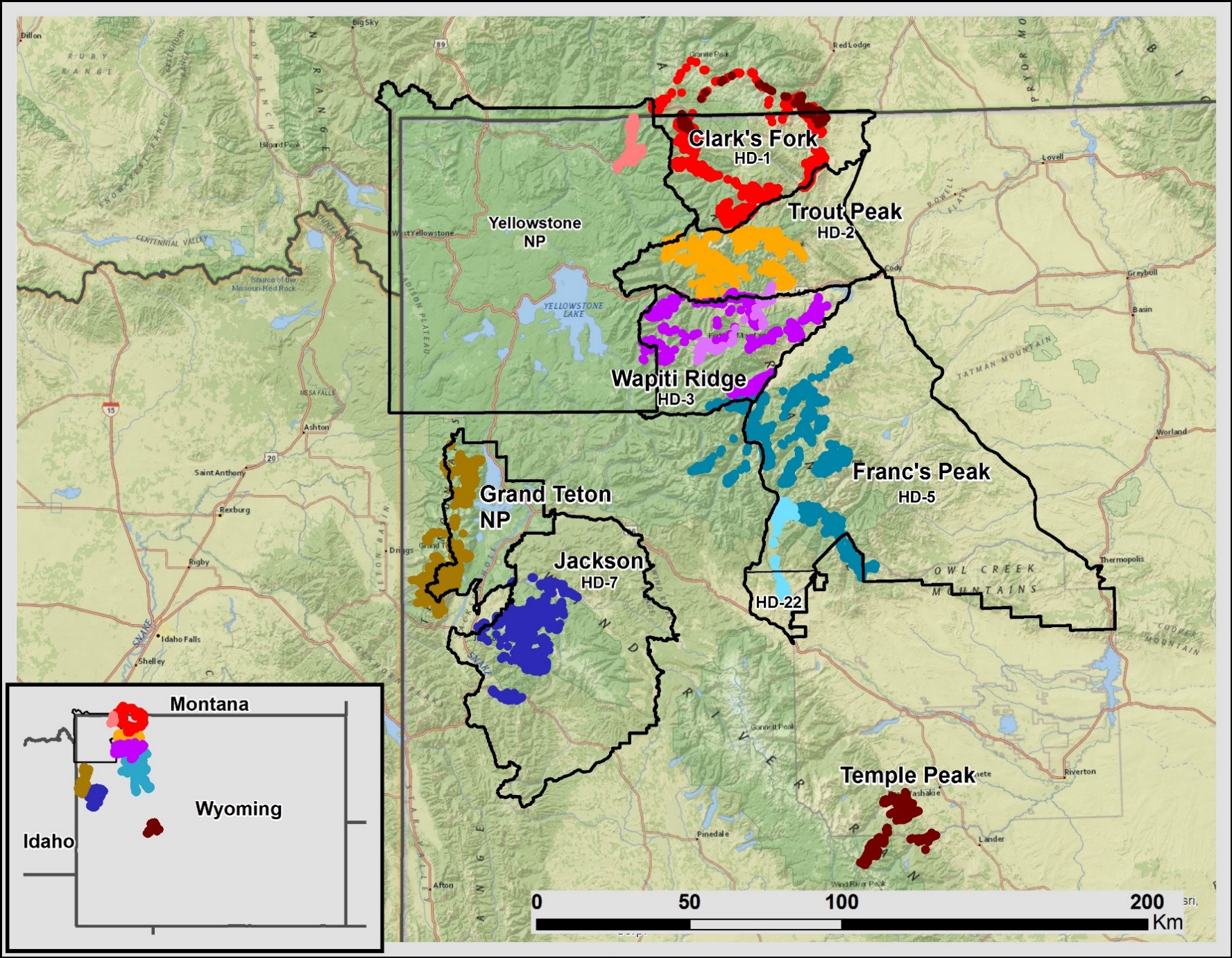


**Fig S1.2** Wyoming herd units. The Clark’s Fork herd unit contained 16 individuals in HD-1 (red), two individuals captured in northeast YNP (pink), and one individual captured just north of the MT-WY boarder (dark red). The Wapiti herd contained six individuals from HD-3 (dark pink) and one individual that was captured in HD-2, but more strongly associated with HD-3 (light pink). The Franc’s Peak herd was an aggregate of HD-5 (blue) and HD-22 (light blue).

# References

Levin, S.A. (1992) The Problem of Pattern and Scale in Ecology: The Robert H. MacArthur Award Lecture. *Ecology*, **73**, 1943–1967.

Wiens, J.A. (1989) Spatial Scaling in Ecology. *Functional Ecology*, **3**, 385–397.
