# Supplementary material for: Characterizing population and individual migration patterns among native and restored bighorn sheep (Ovis canadensis)
Source: Ecol Evol. 2019 Jul 9;9(15):8829–39. doi: 10.1002/ece3.5435 (PMC6686647; doi:10.1002/ece3.5435)
Supplement: Supplementary file 2 [file ECE3-9-8829-s002.docx]

**Appendix S2: Characterizing phenological patterns and topographic heterogeneity among all study populations**

Phenological patterns and landscape heterogeneity are important drivers of migratory behavior in ungulates (Merkle *et al.* 2016; Hsiung *et al.* 2018; Smolko *et al.* 2018). To ensure among population comparisons of migratory patterns were not confounded by differences in phenology or landscape heterogeneity, we characterized the temporal delay in the growing season start date along the elevational gradient in each study population. We defined the available landscape extent based on observed movement metrics rather than rely on hunt units, which often extend far beyond the known distributions of bighorn sheep, or landscape extents determined from GPS locations (i.e., minimum convex polygons) which would have provided a limited characterization of the ‘available’ landscape for resident populations. More specifically, we defined a consistent landscape extent by buffering the centroids of each study population by 30 km - the 95^th^ percent distribution quantile of migration distances among all pooled individuals (Fig S2.1). Given our interest in seasonal movements over potentially broad spatial scales, we content this approach provided the most appropriate landscape extent within which to characterize phenological patterns and topographic heterogeneity.

Using MODIS 250 meter remotely sensed satellite imagery we characterized plant phenology using the start of the growing season metric, which identifies the day of the year when photosynthetic activity significantly increases above winter baselines (USGS EROS Center 2016). For each study population and year (2008-2017) we masked the growing season start date GIS layer with 10 unique elevation rasters which spanned the elevational gradient across all study populations according to 10 equal area bins. Each elevation bin spanned 340 meters and


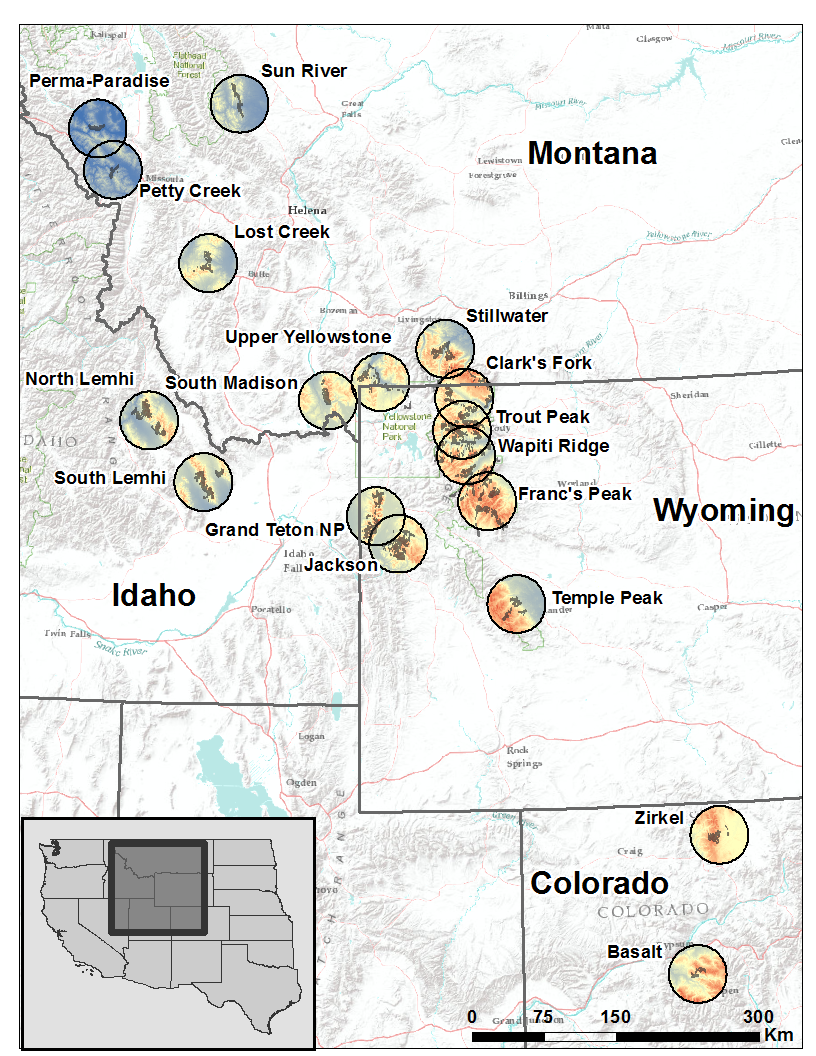


**Fig S2.1** Study population extents used to characterize phenological patterns and topographic heterogeneity, Montana, Wyoming, Idaho, and Colorado, USA, 2008−2017. The relative elevation of each study population is delineated with blue (low) to red (high) coloring. Observed GPS locations are shown for reference (grey points).

was delineated as the 10^th^ to 90^th^ percent distribution quantiles for the elevation values in all study populations (Table S2.1). Lastly, for each study population, year, and elevation bin, we calculated the mean growing season start date. Given the phenological patterns described in other regional study areas (e.g., Jesmer *et al.* 2018), we expected to observe progressively later growing season start dates moving from low to high elevations, indicating a green-wave of vegetation advancing from low to high elevations in each study population. We determined the duration of the green wave as the number of days between the minimum and maximum growing season start dates along the elevation gradient.

**Table S2.1** Minimum and maximum bin values determined by the 10^th^ to 90^th^ percent distribution quantiles across all study populations, Montana, Wyoming, Idaho, and Colorado, USA, 2008−2017.

| **Elevation Bin** | **Min elevation (m)** | **Max elevation (m)** |
| --- | --- | --- |
| 1 | 737 | 1078 |
| 2 | 1079 | 1418 |
| 3 | 1419 | 1758 |
| 4 | 1759 | 2099 |
| 5 | 2010 | 2440 |
| 6 | 2441 | 2780 |
| 7 | 2781 | 3120 |
| 8 | 3121 | 3461 |
| 9 | 3462 | 3802 |
| 10 | 3803 | 4142 |

The mean elevation gradient among all study populations was 1,945 (± 383 SD) meters. Although some study populations occurred in relatively low elevations, all had a progression of growing season start dates which began at low elevations and advanced along the elevation gradient from low to high elevations (Fig S2.2). The growing season advanced over an average of 66 (± 9.84 SD) days among all study populations and was consistent among years (Fig S2.2). Growing season start dates advanced most quickly across the mid-elevation bins in each study area, often resulting in an inverse logistic curve (Fig S2.2). Our results indicate phenological patterns and landscape heterogeneity were similar among the study populations and that restored, augmented, and native populations all had the presence of green waves and similar variation in landscape topography (Fig S2.2).


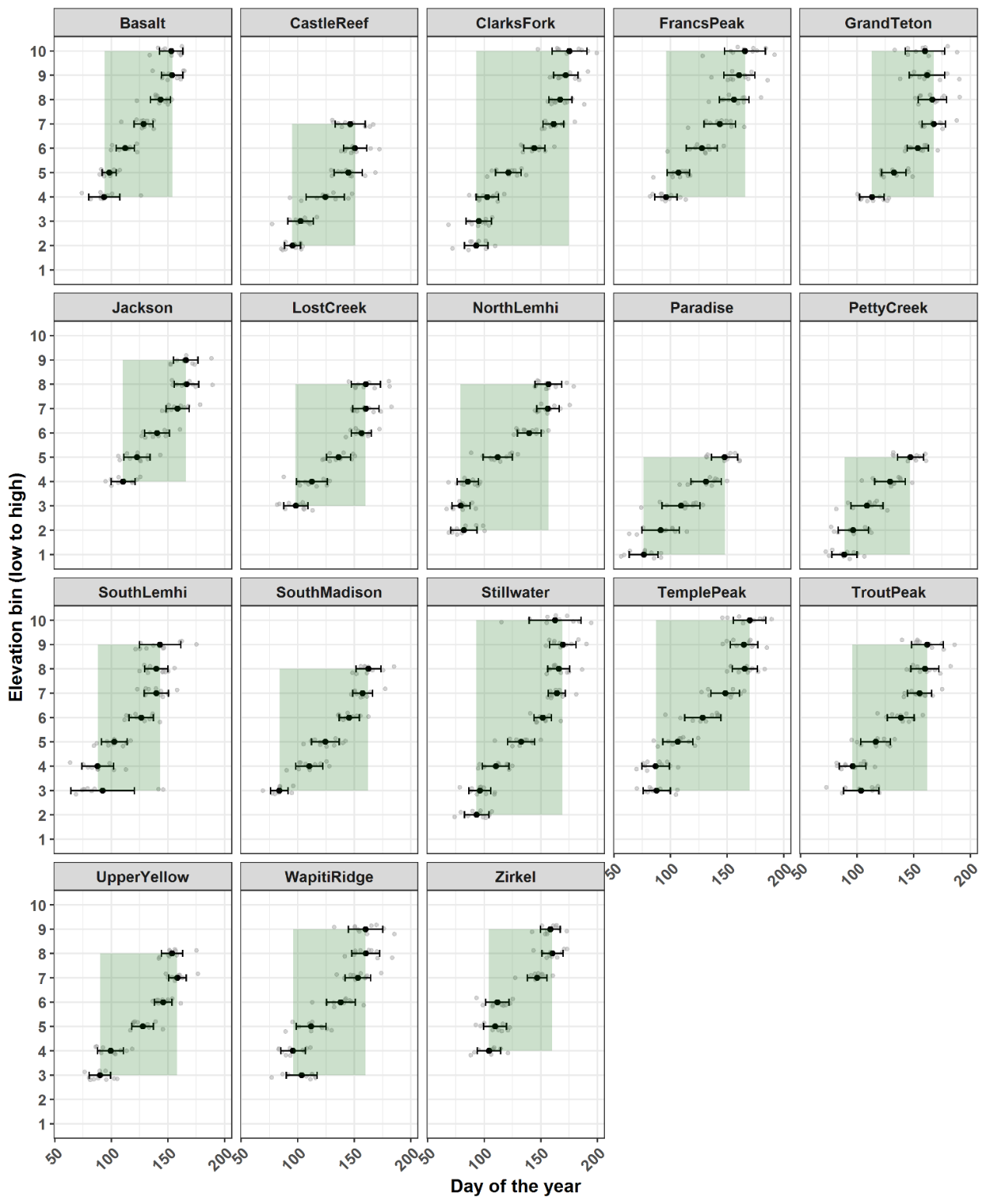


**Fig S2.2** Mean growing season start dates (± SD) for each elevation bin and study population, Montana, Wyoming, Idaho, and Colorado, USA, 2008−2017. Elevation bins transition from low (1) to high (10) elevations on the y-axis. The yearly (2008-2017) growing season start dates for each elevation bin are also shown (light grey points). The green shading represents the temporal duration and vertical gradient over which the green wave advanced.

**References**

Hsiung, A.C., Boyle, W.A., Cooper, R.J. & Chandler, R.B. (2018) Altitudinal migration: ecological drivers, knowledge gaps, and conservation implications. *Biological Reviews,* **93**, 2049–2070.

Jesmer, B.R., Merkle, J.A., Goheen, J.R., Aikens, E.O., Beck, J.L., Courtemanch, A.B., Hurley, M.A., McWhirter, D.E., Miyasaki, H.M., Monteith, K.L. & Kauffman, M.J. (2018) Is ungulate migration culturally transmitted? Evidence of social learning from translocated animals. *Science*, **361**, 1023–1025.

Merkle, J.A., Monteith, K.L., Aikens, E.O., Hayes, M.M., Hersey, K.R., Middleton, A.D., Oates, B.A., Sawyer, H., Scurlock, B.M. & Kauffman, M.J. (2016) Large herbivores surf waves of green-up during spring. *Proc. R. Soc. B*, **283**, 20160456.

Smolko, P., Kropil, R., Pataky, T., Veselovská, A. & Merrill, E. (2018) Why do migrants move downhill? The effects of increasing predation and density on red deer altitudinal migration in temperate Carpathian forests. *Mammal Research*, **63**, 297–305.

USGS EROS Center. 2016. Remote sensing phenology. United States Geological Survey Earth Resources Observation and Science Center, Sioux Falls, South Dakota, USA. http://phenology.cr.usgs.gov/
