# Supplementary material for: Characterizing population and individual migration patterns among native and restored bighorn sheep (Ovis canadensis)
Source: Ecol Evol. 2019 Jul 9;9(15):8829–39. doi: 10.1002/ece3.5435 (PMC6686647; doi:10.1002/ece3.5435)
Supplement: Supplementary file 3 [file ECE3-9-8829-s003.docx]

**Appendix S3: Population attributes and sample sizes**

*Population size*

The most recent estimates of population sizes were provided by agency management biologists and determined from local knowledge, minimum counts, and recent trends, and were varied across the three management histories (Table 1; Fig S3.1). Restored and augmented populations had an average estimate of 139 and 142 individuals, respectively, while native populations had an average estimate of 551 individuals (Fig S3.1). While density can be an important attribute in migratory propensity (i.e., Mysterud *et al.* 2011) we were unable to estimate seasonal densities across our study populations. Although native populations were more abundant, the native population units and seasonal ranges were often larger than restored or augmented populations. Moreover, native populations often had a subpopulation structure with multiple aggregates of individuals within the spatial extent of the population units. Given the variable nature of subpopulation numbers and locations among study areas and years, we were unable to define local seasonal ranges for the subpopulations. As a result, estimates of abundance were aggregated at the population level, and do not represent local densities on summer or winter ranges. While the population estimates provide a measure of relative abundance within and among management histories, they do not equate with relative seasonal densities.


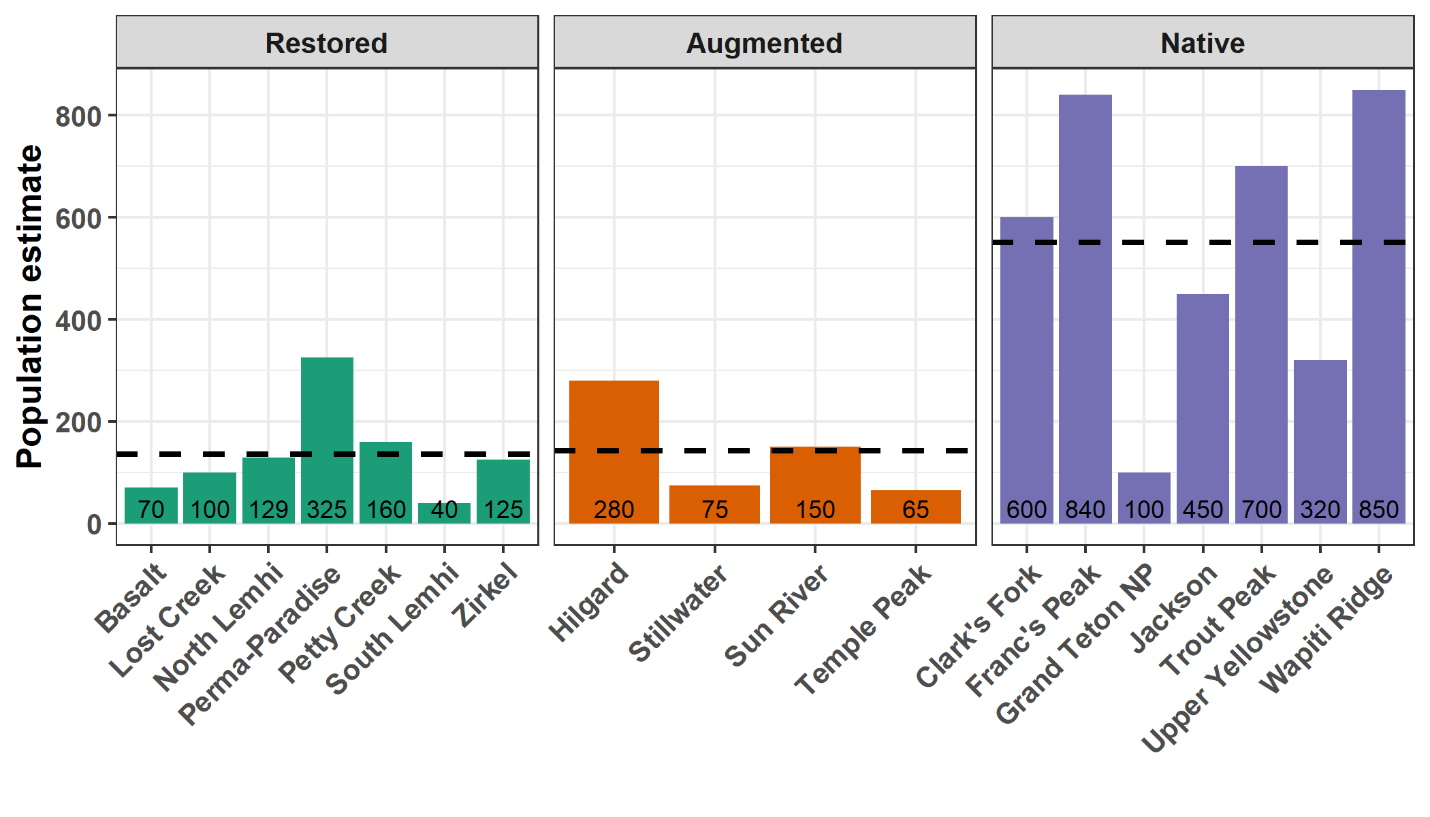


**Fig S3.1** Population estimates from agency management biologists determined from local knowledge, minimum counts, and recent trends for all study areas, Montana, Wyoming, Idaho, and Colorado, USA, 2008−2017. The average population estimate for each management history is shown with a dashed line. Restored and augmented populations had average population estimates of 139 and 142 individuals, respectively. The average estimate for native populations was 551 individuals. Estimates for each population are shown in the bottom of the respective bar.

*Sample size*

Our sample of instrumented individuals was relatively consistent across the three management histories and ranged from 7 to 19 individuals (Table 1; Fig S3.2). The average sample among restored, augmented, and native populations was 10 (± 3.3 SD), 12 (± 2.94 SD), and 13 (± 4.27 SD) individuals, respectively (Fig S3.2). Although sample sizes were slightly larger in native populations, our results were robust to the different sample sizes in each population (Appendix S4).


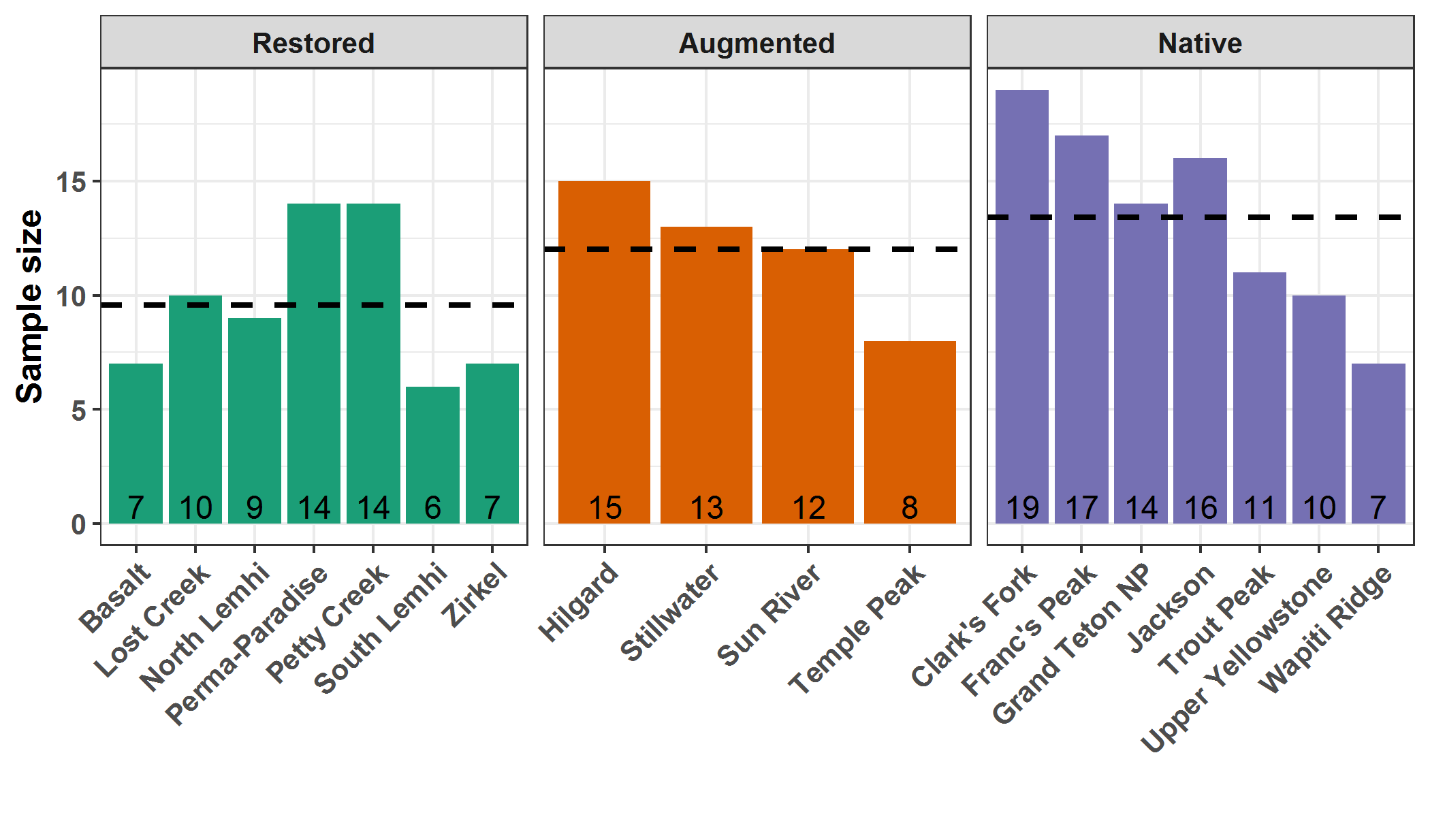


**Fig S3.2** Number of instrumented individuals in each study population, Montana, Wyoming, Idaho, and Colorado, USA, 2008−2017. The average sample size for each management history is shown with a dashed line (Restored = 10, Augmented = 12, Native = 13). Sample sizes for each population are shown in the bottom of the respective bar.

*Translocation histories*

Translocation histories were varied among restored and augmented populations. On average, augmented populations received more translocated individuals and had more translocation events than restored populations, although this pattern was largely driven by the Hilgard and Temple Peak populations, which received 69 and 165 individuals through three and seven translocation events, respectively (Figs S3.3 and S3.4). Restored populations received an average of 33 individuals through two translocation events (Figs S3.3 and S3.4).


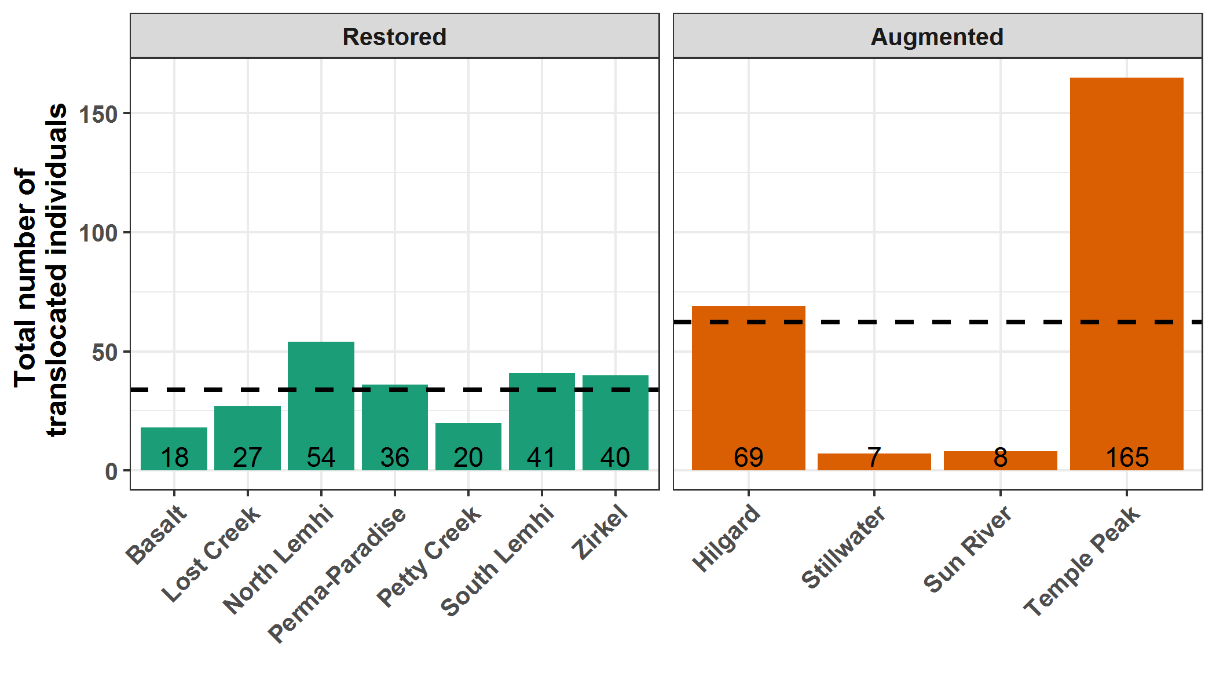


**Fig S3.3** Total number of bighorn sheep translocated into each restored and augmented study population, Montana, Wyoming, Idaho, and Colorado, USA, 2008−2017. The average number of translocations for each management history is shown with a dashed line (Restored = 33, Augmented = 62). The number of animals translocated into each population are shown in the bottom of the respective bar.


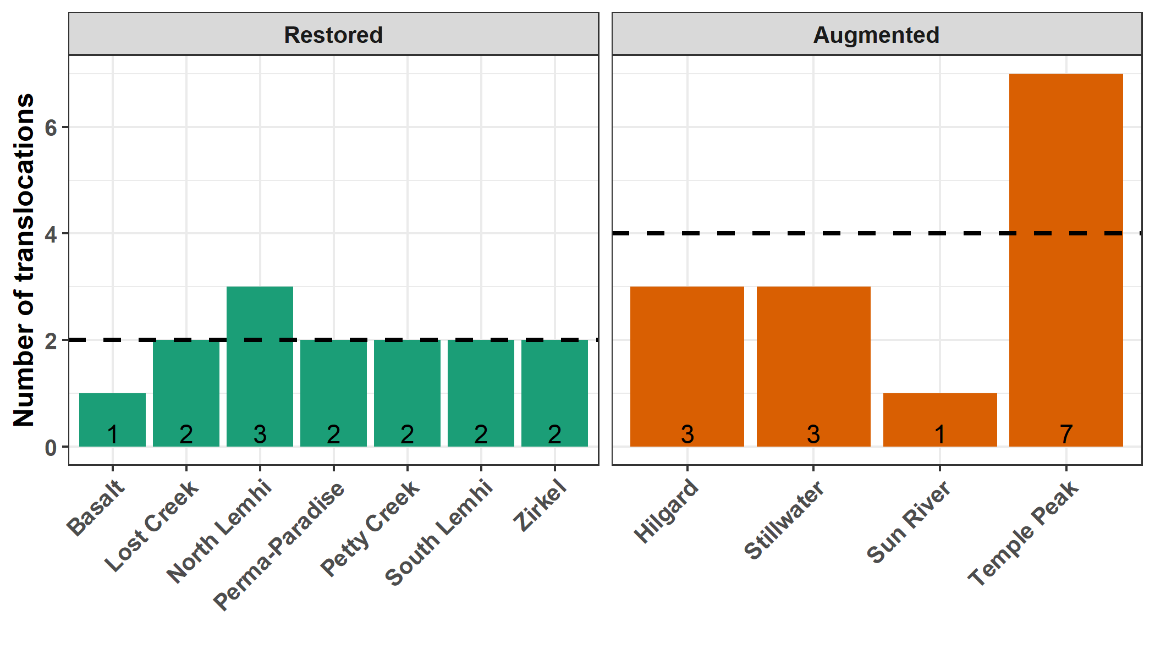


**Fig S3.4** The number of translocation events for each restored and augmented study population, Montana, Wyoming, Idaho, and Colorado, USA, 2008−2017. The average number of translocation events for each management history is shown with a dashed line (Restored = 2, Augmented = 4). The number of translocation events for each population are shown in the bottom of the respective bar.

The number of years since animals were initially translocated in restored and augmented populations was an important population characteristic. Within the context of learned migration behaviors in bighorn sheep, translocated animals that have had a relatively long period within the new landscape were expected to have a greater migratory propensity when compared to animals that were recently translocated (Jesmer *et al.* 2018). Restored and augmented populations had an average of 34 (SD = 12.7) and 46 (SD = 12.3) years, respectively, since the initial translocation (Fig S3.5). The 12 year difference between restored and augmented populations was likely negligible as migratory behavior builds upon generational knowledge that is gained over the course of many decades (Jesmer *et al.* 2018).


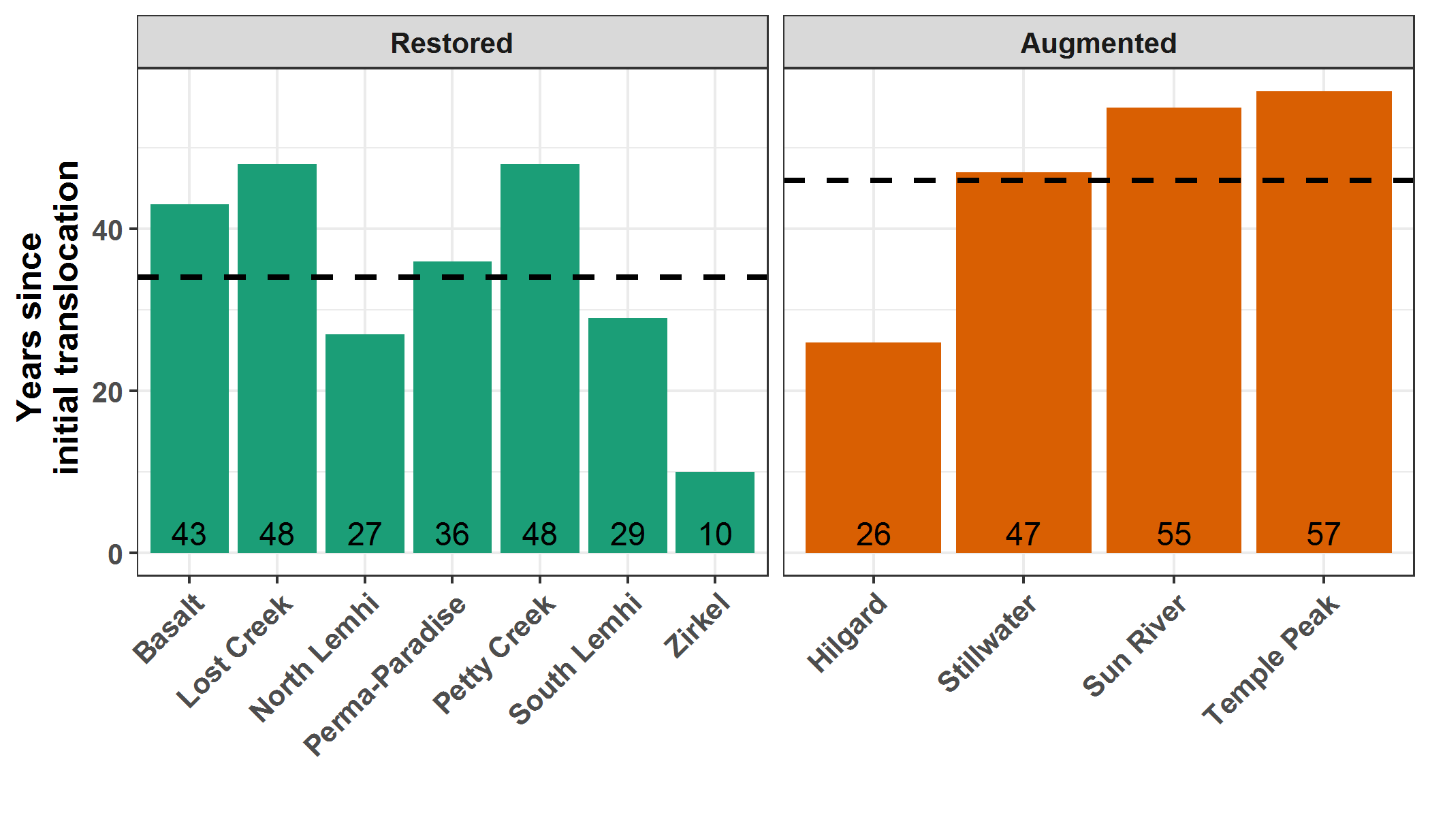


**Fig S3.5** The number of years since the initial translocation for each restored and augmented study population, Montana, Wyoming, Idaho, and Colorado, USA, 2008−2017. The average number of years for each management history is shown with a dashed line (Restored = 34, Augmented = 46). The number of years since the initial translocation for each population are shown in the bottom of the respective bar.

Lastly, the migratory behaviors of the source population were an additional population attribute of interest. While the migratory tendencies of translocated individuals are not generally known, migratory patterns of source populations are often documented through historic reports, VHF monitoring, or GPS collar data. The varying sources of information can result in differing degrees of knowledge across source populations. Nonetheless, the use of migratory or partially migratory source populations was the most common translocation strategy (Fig S3.6). Translocations from both resident and migratory sources were used in the Hilgard and Petty Creek populations. Perma-Paradise was the only population restored exclusively with resident individuals (Fig S3.6). Given the limited number of resident source populations, we were unable to draw definitive conclusions regarding the effect of source population strategy on contemporary migratory diversity. However, it should be noted that with the exception of Petty Creek, all populations that were restored with individuals from migratory sources had a migratory component (Fig S3.6; Appendix S5). In contrast, Perma-Paradise was the only population that was exclusively restored from resident sources and was a contemporary resident population (Fig S3.6; Appendix S5). Given the observed benefits to migratory behavior in bolstering restoration success (Singer, Papouchis & Symonds 2000), we suggest using migratory source populations unless area specific management priorities favor a resident behavior.


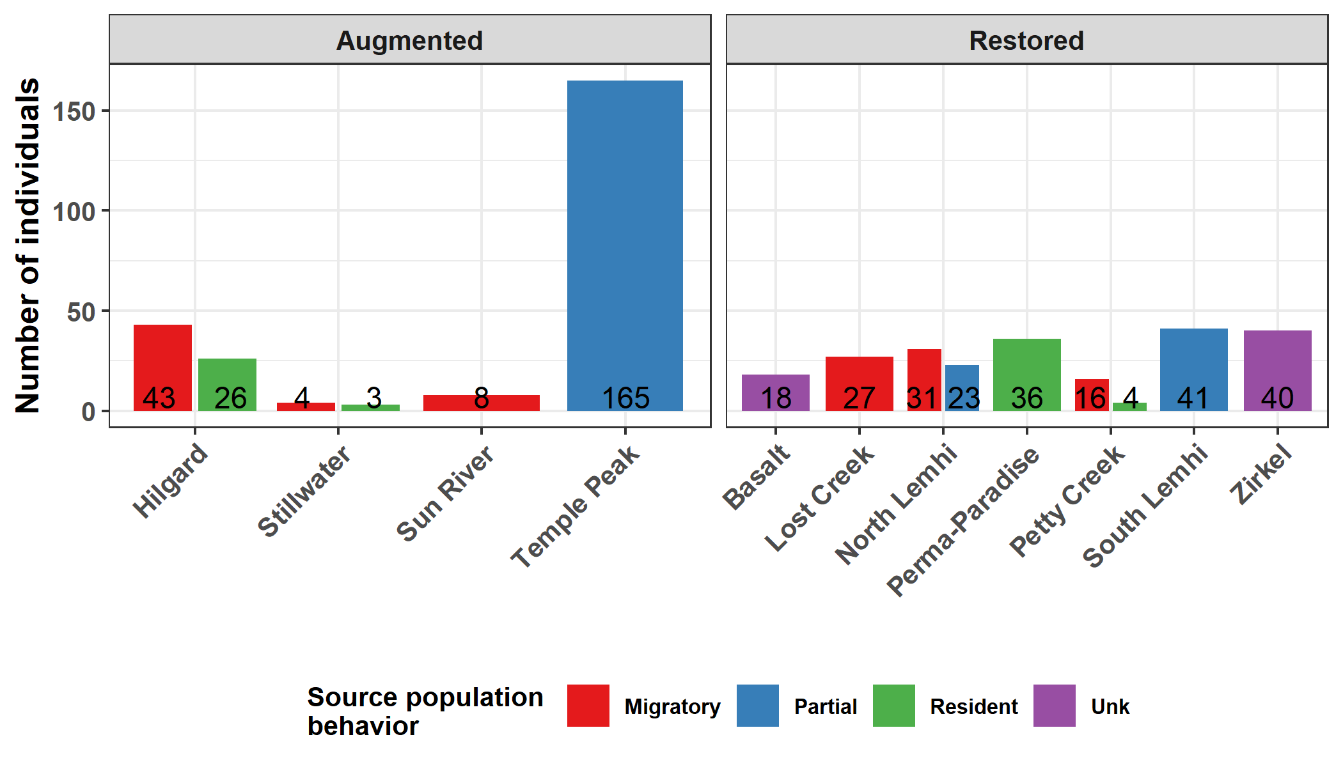


**Fig S3.6** The number of individuals translocated into each study population from migratory (red), partially migratory (blue), resident (green), and unknown (purple) source populations, Montana, Wyoming, Idaho, and Colorado, USA, 2008−2017.

**References**

Jesmer, B.R., Merkle, J.A., Goheen, J.R., Aikens, E.O., Beck, J.L., Courtemanch, A.B., Hurley, M.A., McWhirter, D.E., Miyasaki, H.M., Monteith, K.L. & Kauffman, M.J. (2018) Is ungulate migration culturally transmitted? Evidence of social learning from translocated animals. *Science*, **361**, 1023–1025.

Mysterud Atle, Loe Leif Egil, Zimmermann Barbara, Bischof Richard, Veiberg Vebjørn & Meisingset Erling. (2011) Partial migration in expanding red deer populations at northern latitudes – a role for density dependence? *Oikos*, **120**, 1817–1825.

Singer, F.J., Papouchis, C.M. & Symonds, K.K. (2000) Translocations as a tool for restoring populations of bighorn sheep. *Restoration Ecology*, **8**, 6–13.
