# Supplementary material for: Characterizing population and individual migration patterns among native and restored bighorn sheep (Ovis canadensis)
Source: Ecol Evol. 2019 Jul 9;9(15):8829–39. doi: 10.1002/ece3.5435 (PMC6686647; doi:10.1002/ece3.5435)
Supplement: Supplementary file 4 [file ECE3-9-8829-s004.docx]

**Appendix S4: Simulation to evaluate the effect of sample size on the observed variation in elevational and geographic migration distances**

Although the ranges of sample sizes were relatively consistent among the three management histories (restored, augmented, and native), on average we instrumented slightly more individuals per population in native populations than in restored or augmented populations [Appendix S3; Restored = 10 (± 3.3 SD), Augmented = 12 (± 2.94 SD), Native = 13 (± 4.27 SD)]. The modest disparities in sample sizes among the three management histories was the result of regional funding allocations, collar failures, and diminishing returns in the information gained with continued deployments in some restored populations. For example, in instances where populations were small or had little variation among individuals, a relatively modest number of collar deployments accurately described population-level migratory patterns. Rather than continue to deploy collars in these situations, instrumentation efforts were abbreviated.

To ensure the larger sample sizes in the native populations were not the source of increased variation in the observed elevation and geographic migratory distances, we conducted a bootstrap simulation to resample and characterize population-level variation using a consistent sample size across all study populations. We conducted 1000 iterations in which we used the minimum sample of six individuals per population to characterize the variation in population-level migration distances (elevation and geographic) across restored, augmented and native management histories. More specifically, for each iteration we randomly sampled six individuals from each population and calculated the difference between the 90^th^ and 10^th^ percent distribution quantiles for elevational and geographic migration distance. Each iteration resulted in a single point for each population representing the range of variation along the elevation and geographic axes as calculated from six individuals. We then aggregated the results according to the management history to compare population-level variation among restored, augmented and native management histories.

The observed differences in population-level variation with the consistent sample size of six individuals in each study population was consistent with our results from the complete dataset. Across all simulations the mean geographic difference between the 90^th^ and 10^th^ percent quantiles was notably higher for native populations (19 ± 10.4 km) than for restored (5.5 ± 3.21 km) or augmented (8.9 ± 5.22 km) populations. Although more modest, the mean elevational difference was also consistently higher for native (618 ± 233 m) populations, than for restored (388 ± 267 m) or augmented (524 ± 465 m) populations. The simulation results mirror those described with the full dataset and show strikingly less variation in migration distances among individuals in restored and augmented populations than in native populations (Fig S4.1). These results support our use of the complete dataset and indicate that our observed differences in migration distances are robust to the slight differences in sample sizes across restored, augmented, and native populations of bighorn sheep.


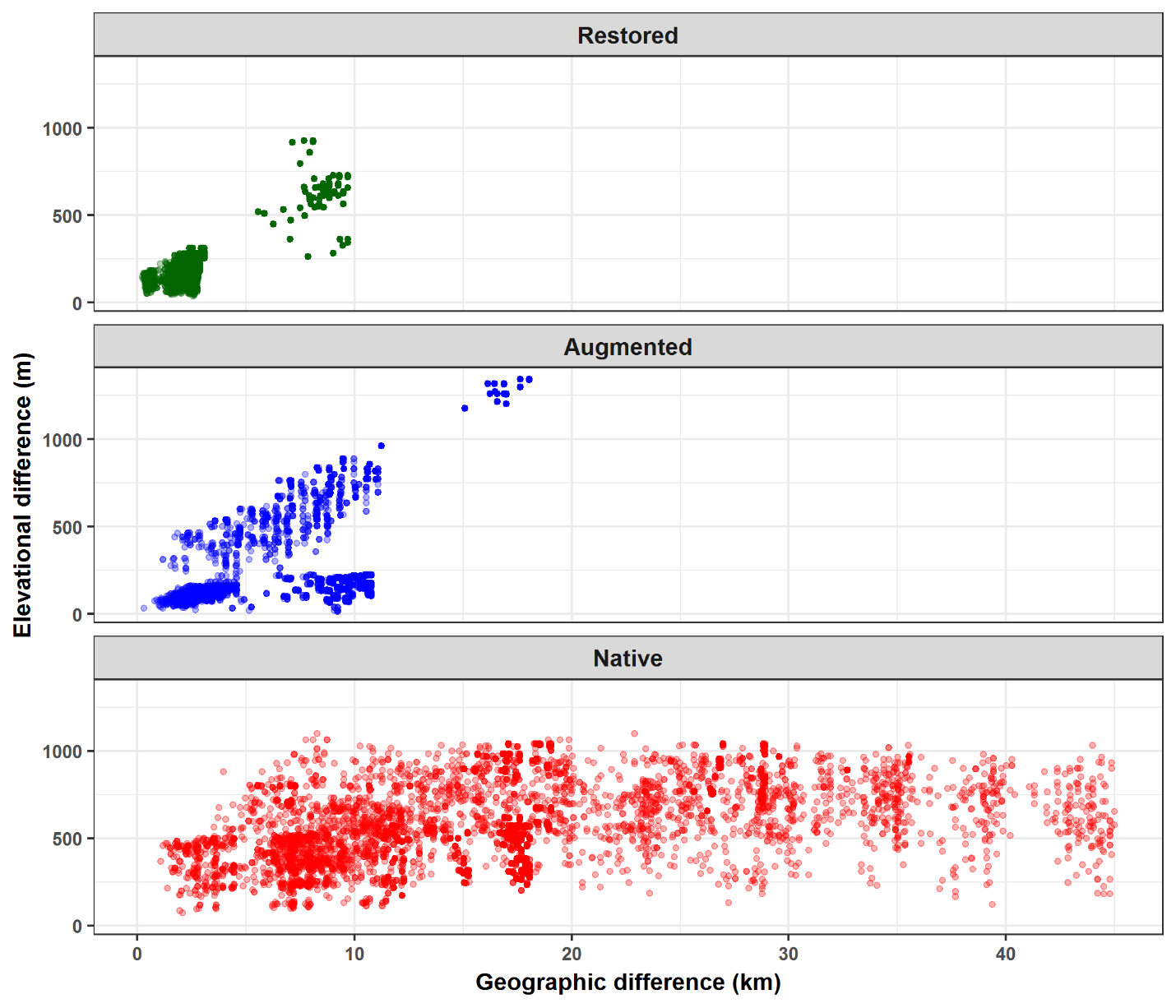


**Fig S5.1** Simulation results from 1000 bootstrap iterations in which six individuals were randomly sampled from each study population to characterize population-level variation in migratory distance with a constant sample size, Montana, Wyoming, Idaho, and Colorado, USA, 2008−2017. Restored (green), augmented (blue), and native (red) populations are shown in the top, middle, and lower panels. The x- and y-axis represent the difference between the 90^th^ and 10^th^ percent distribution quantiles for geographic and elevational migration distances, respectively.
