# Supplementary material for: Characterizing population and individual migration patterns among native and restored bighorn sheep (Ovis canadensis)
Source: Ecol Evol. 2019 Jul 9;9(15):8829–39. doi: 10.1002/ece3.5435 (PMC6686647; doi:10.1002/ece3.5435)
Supplement: Supplementary file 5 [file ECE3-9-8829-s005.docx]

**Appendix S5: Individual migration characterizations**

The individual migration characterizations for restored (Fig S5.1), augmented (Fig S5.2), and native (Fig S5.3) populations help to show the patterns driving population-level variability. Within restored and augmented populations, population-level variability was driven by the resident and migratory behaviors of partially migratory population as seen in Basalt (Fig S5.1) and Temple Peak (S5.2). In contrast, native populations had a continuum of individual movements with respect to elevational and geographic distances, representing a portfolio of seasonal migration patterns (Fig S5.3).

**
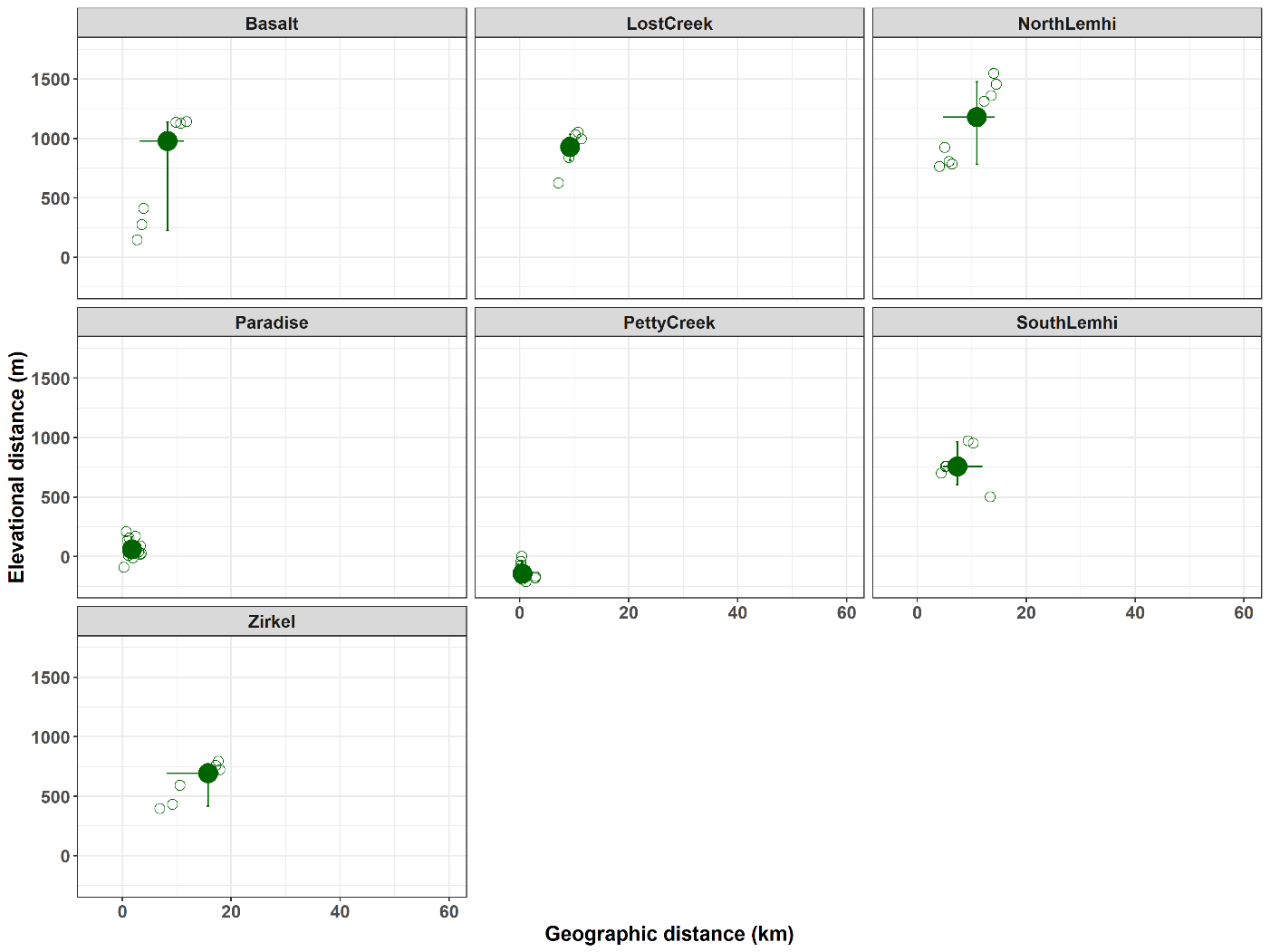
**

**Fig S5.1** Restored herd-level (solid circles) and individual (open circles) migration characterizations. Lines represent the herd-level 10^th^ and 90^th^ percent distribution quantiles for each axis.

**
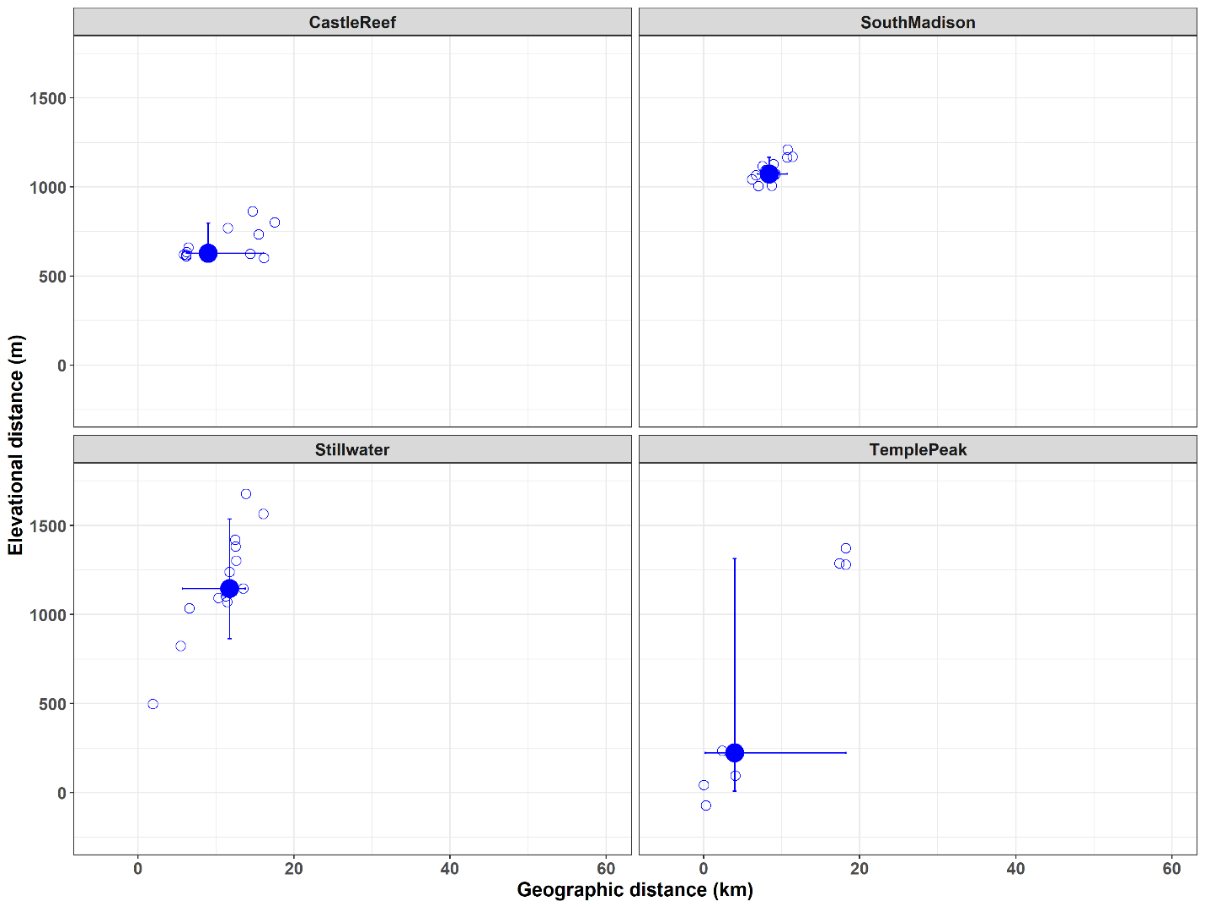
**

**Fig S5.2** Augmented herd-level (solid circles) and individual (open circles) migration characterizations. Lines represent the herd-level 10^th^ and 90^th^ percent distribution quantiles for each axis.


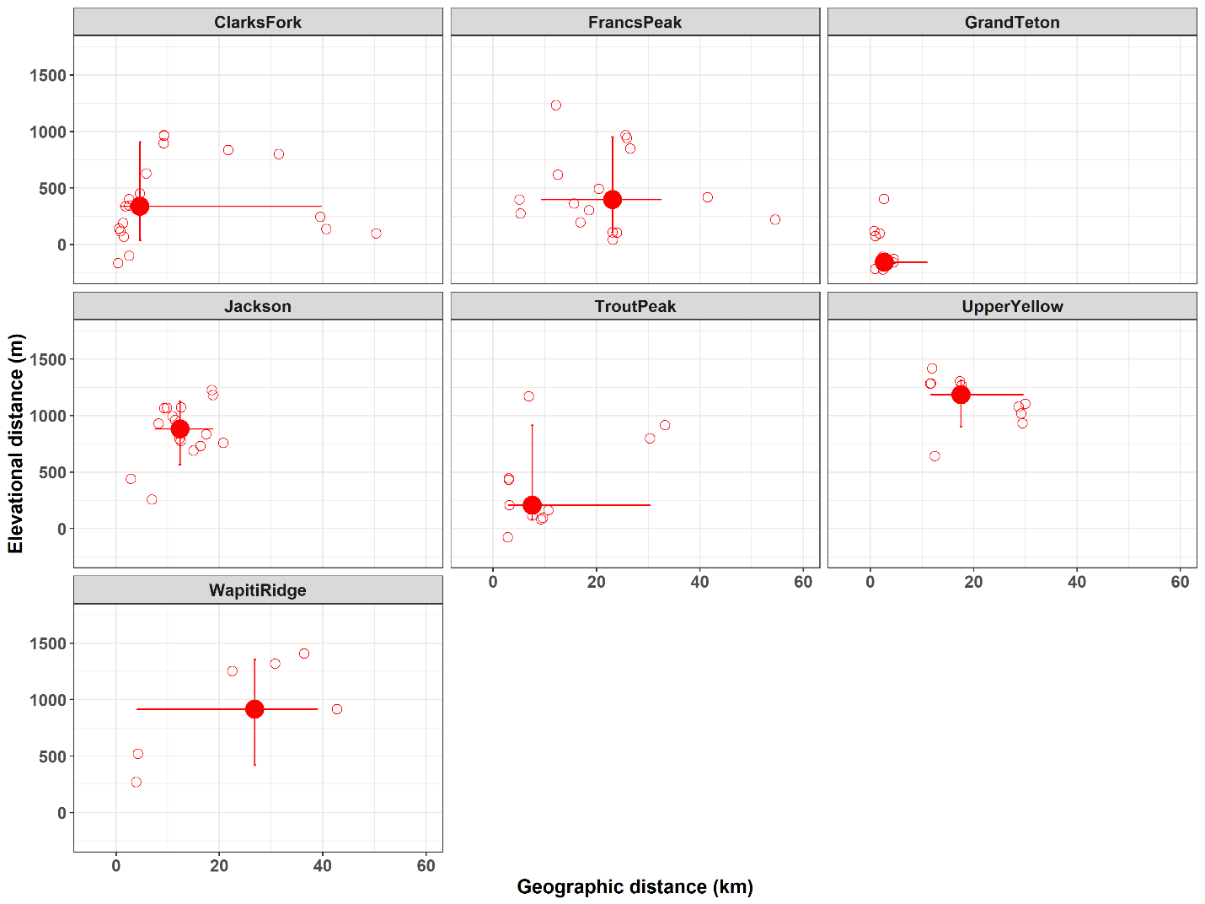


**Fig S5.3** Native herd-level (solid circles) and individual (open circles) migration characterizations. Lines represent the herd-level 10^th^ and 90^th^ percent distribution quantiles for each axis.
